# Understanding the Mechanism of Nontraditional Zeolite Synthesis Using *In Situ* Nuclear Magnetic Resonance Spectroscopy and X‑ray Diffraction

**DOI:** 10.1021/jacs.5c17807

**Published:** 2025-12-22

**Authors:** Nicole L. Kelly, Emma A. L. Borthwick, Gaynor B. Lawrence, Paul S. Wheatley, Arosha A. K. Karunathilake, Oxana V. Magdysyuk, David C. Lloyd, Colan E. Hughes, Kenneth D. M. Harris, Russell E. Morris, Sharon E. Ashbrook

**Affiliations:** 1 School of Chemistry, EaStCHEM and Centre of Magnetic Resonance, 7486 University of St Andrews, North Haugh, St Andrews KY16 9ST, U.K.; 2 School of Chemistry, 2112 Cardiff University, Park Place, Cardiff CF10 3AT, U.K.

## Abstract

*In situ* solid-state nuclear magnetic resonance (NMR) spectroscopy and *in situ* powder X-ray diffraction (PXRD) experiments are used to develop mechanistic insights into the disassembly and organization steps of nontraditional zeolite synthesis using the ADOR (Assembly, Disassembly, Organization, Reassembly) process. The work focuses on the reaction of the germanosilicate zeolite UTL to form two ADOR intermediates: IPC-1P on reaction with water and IPC-2P on reaction with aqueous HCl. The changes in the local structure on reaction with water can be modeled as one overall disassembly process, but the long-range changes, as measured by changes in interlayer spacing determined by XRD, indicate multiple stages of the reaction as the layer structure develops. For the reaction with aqueous acid, the local changes are modeled with two processes: a disassembly and an interlayer rearrangement (organization step). However, only one major stage of change is seen in the XRD measurements. The new details revealed by the *in situ* studies demonstrate that both local (probed by NMR spectroscopy) and long-range (probed by XRD) changes to the structure are required to truly understand how the reaction proceeds. The results provide new insights into the relative kinetics of the different processes involved in the reactions under different conditions and reveal new features such as staging in the layer stacking changes in the organization step.

## Graphical abstract

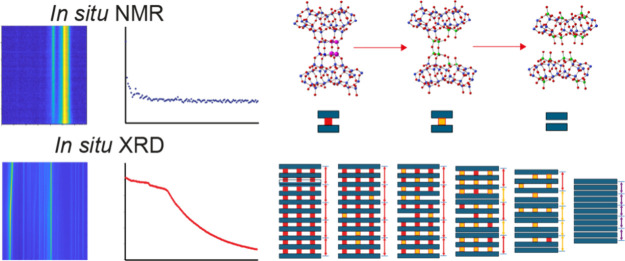

## Introduction

The ADOR (Assembly, Disassembly, Organization, Reassembly) process has generated significant interest as an alternative, nontraditional, method for the synthesis of high silica zeolites, exploiting inherent weakness within the structure of a pre-existing “parent” material.^1–4^ Although silicate-based zeolites are one of the most important classes of porous materials, with industrial applications as catalysts and adsorbents,^5^ the hydrothermal methods^6,7^ commonly used for their synthesis can be difficult to control, and many topologies that are theoretically possible appear to be experimentally inaccessible using this approach.^8^ Therefore, the ability to target zeolites with new topologies and specific pore sizes in a more controllable manner has been the challenging aim of new synthetic methods in the past few years.^8–14^

In the ADOR approach, a hydrolytically sensitive dopant element (such as Ge) is incorporated into a preprepared parent zeolite at specific positions within the framework (the assembly step).^1–4^ Germanium has been used for several decades as an additive in zeolite synthesis and there is overwhelming evidence that it is preferentially incorporated into the double four ring (d4r) units in the final zeolite. The evidence for this preferential incorporation comes from both experimental^10,15^ and theoretical^16,17^ studies and seems particularly strong for zeolite UTL. The specific location of Ge within the d4r units enables a regioselective removal of the dopant under aqueous conditions, giving a controlled disassembly to generate stable zeolitic intermediates, from which new “daughter” zeolites can be produced once the intermediates are organized in a suitable manner to allow reassembly to occur. This organization can simply involve the relative rearrangement of the existing building units, the reintercalation of silica from solution or the deliberate addition of organizing agents, with reassembly, a topological condensation usually at higher temperatures, then giving rise to the final fully condensed framework. The ADOR process has been shown to be sensitive to the exact experimental conditions used, with changes in framework composition, temperature, pH, solvent, pressure and scale leading to intermediates and products with varied compositions, structures and reactivity.^1–4^ This versatility enables a range of daughter materials to be produced from a single parent material by judicious choice of the reaction conditions. As an example, Figure 1 shows a schematic of the ADOR process applied to a Ge-UTL^18,19^ parent material. The Ge is preferentially located within the d4r between the silica-rich zeolitic layers, and its selective removal during hydrolysis gives rise to partially connected or layered intermediates such as IPC-2P* and IPC-1P.^1^ IPC-1P can be reassembled directly to form IPC-4 or can undergo further rearrangement to form intermediates such as IPC-6P or IPC-2P, which can be calcined to give zeolites IPC-6 and IPC-2, respectively.^20–25^

**Figure 1:**
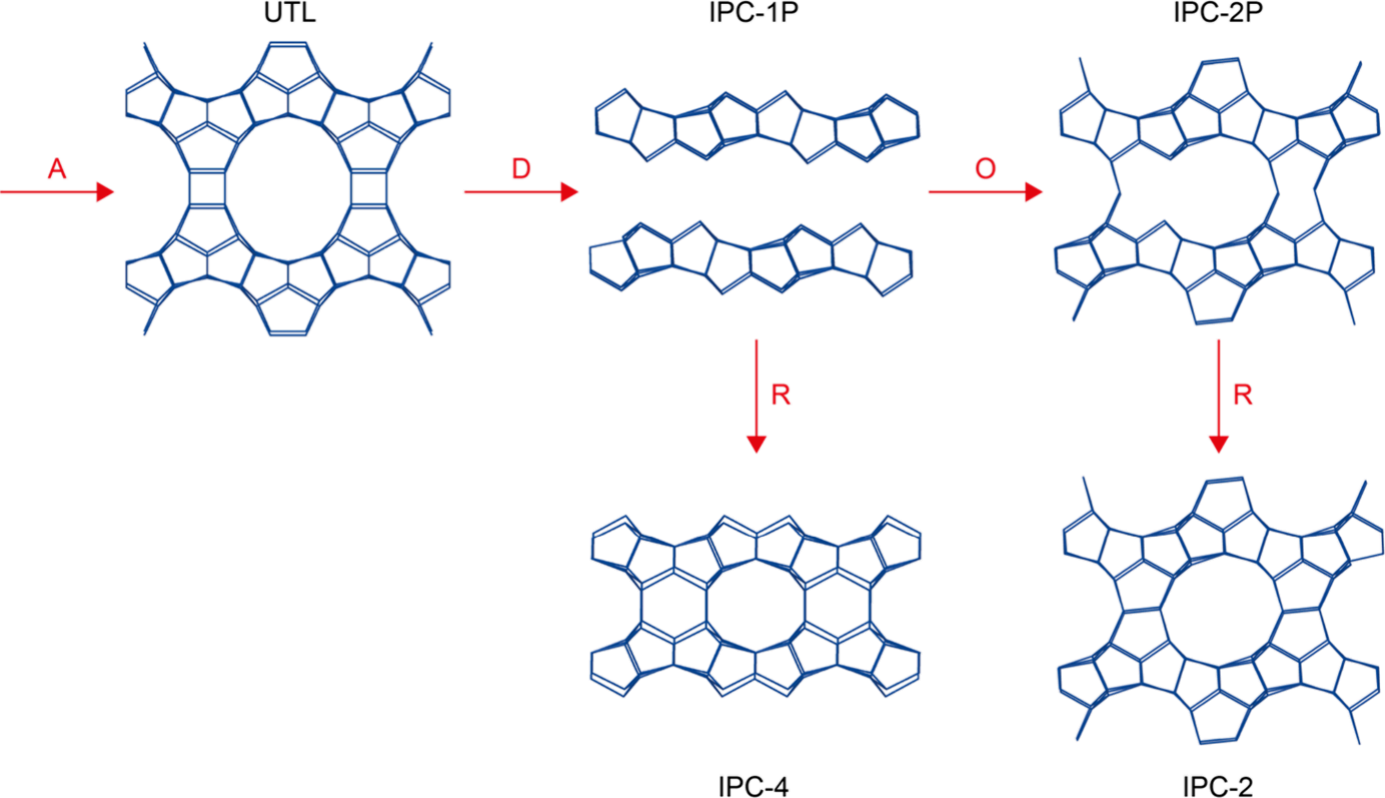
Schematic showing an example of the ADOR process applied to Ge-UTL. Selective disassembly leads to IPC-1P, which upon heating gives IPC-4. Further organization of IPC-1P leads to IPC-2P, which can be reassembled to give IPC-2. In this depiction of the structure, the centers of the TO_4_ tetrahedra (T = Si or Ge) are linked by the lines, and the oxygen atoms are not shown.

Despite its significant success, the exact mechanism by which the ADOR reaction proceeds, and how this varies with different experimental conditions, is still poorly understood. However, by analogy with the extensive work on the chemistry of silica and silicates, we can predict that there are several possible reactions.^26^ The low hydrolytic stability of Ge–O–T bonds (where T = Ge or Si) means that this process occurs under any aqueous-based conditions. The condensation of silanol units to form Si–O–T linkages is usually promoted using either acid or base catalysis. However, the situation is complicated in the case of base catalysis as this also promotes depolymerization of silica. In the ADOR process we want to keep the majority of the silica regions of the starting zeolite intact and base catalysis is not used. Powder X-ray diffraction (PXRD) has been used to characterize the starting materials and final products, typically by considering the position of the {200} reflection, which can be related directly to the spacing of the zeolitic layers.^2^ This approach has also been employed to follow the progress of an ADOR reaction (of Ge-UTL with Si/Ge ≈ 4.4) by sampling the material present at various stages of a reaction carried out in water at temperatures between 70 and 100 °C.^23^ Although XRD provides useful information on the *d* spacings in crystalline materials, the long-range order is often partially or completely lost in the intermediate materials formed during the ADOR reaction, and further insight can be obtained by using techniques that are sensitive to the local structural changes, such as solid-state NMR spectroscopy.^27–29^ This technique has been widely applied, often in combination with DFT calculations, to understand local structure, disorder and dynamics in a range of porous solids.^30–35^ The majority of NMR studies of the ADOR process and its products have exploited ^29^Si (I = 1/2) NMR spectroscopy.^1,20,22–24^ The relative proportions of different *Q^n^* Si species present (where n denotes the number of linkages a tetrahedral Si forms through O to other Si species) is characteristic of a specific material. By monitoring the variation in the relative proportions of the *Q^n^* Si species, subtle changes in the local structure may be established. ^17^O (I = 5/2) NMR spectroscopy has also been used to follow the ADOR reaction of Ge-UTL with a 6 M solution of HCl (40% enriched in H_2_^17^O)^21^ and with H_2_^17^O (20% enriched in H_2_^17^O),^24^ which revealed the dynamic behavior of the zeolitic layers (where ^17^O was also incorporated during the reaction). Subsequent work surprisingly showed a similar lability of the framework Si–O–Si bonds in aluminosilicate zeolites at room temperature when in contact with water,^36,37^ demonstrating the possible complexity of zeolite reactivity in aqueous conditions.

Understanding the changes to the local structure that take place during the ADOR process, determining how/if these drive the changes in the average spacing of the silica-rich zeolitic layers and establishing how this varies with experimental conditions are key to gaining insight, and ultimately better control, of the mechanistic steps that take place. The ideal approach is to follow these reactions *in situ*, with different analytical measurements made in real time during the reaction. One of the best options would be to combine the complementary information that can be derived from XRD (i.e., on the long-range order and average structure) and from NMR spectroscopy (which can simultaneously probe the local environments in both liquid- and solid-state components present in the reaction, using techniques such as CLASSIC^38,39^ or SASSY^40^ NMR experiments). The small size of the NMR rotor restricts the scale of the reaction that can be carried out (e.g., 15 mg of zeolite is typically reacted with 15 μL of hydrolyzing solution) and when combined with the low natural abundance of ^29^Si (∼4.7%), this results in poor spectral sensitivity. In principle, this can be overcome through ^29^Si isotopic enrichment of the parent UTL zeolite (and enrichment of any silicon-containing material added during the organization step as shown in previous work), allowing the acquisition of spectra with sufficient sensitivity at time intervals spaced such that the progress of the reaction can be followed in detail.^20,41^

In this work we combine *in situ* NMR spectroscopy and *in situ* PXRD to understand the mechanism of the ADORable hydrolysis of Ge-UTL at temperatures between 20 and 80 °C. Care was taken to ensure that similar reaction conditions (e.g., scale, concentrations, etc.) were used for the NMR and XRD experiments, ensuring that they were as comparable as possible. The only significant difference in the experiments is the rotation rate of the sample; for the NMR experiments this is 5 kHz while in the XRD experiments it is only a few Hz. While the faster spinning in the NMR experiment could, in principle, have an effect on mixing of the reagents, this is likely to be a small effect at this scale.^42^ The reactions have been studied in water and under acidic conditions to understand the effect of pH on the reaction rate and the mechanistic pathway followed. The combination of these two techniques (supported by *ex situ* microscopy measurements) provides a powerful approach, where the interdependency of the changes to the local and average structure can be elucidated, enabling new insight into this complex but versatile method for the synthesis of new zeolite frameworks.

## Methodology

### Synthesis of ^29^Si-Enriched Ge-UTL

^29^Si-enriched Ge-UTL was prepared as described in detail in refs. 20 and 41 using (6R,10S)-6,10-dimethyl-5-azoniaspiro­[4,5]­decane hydroxide as an SDA with both natural abundance (2.094 g) and 99% ^29^Si-enriched (0.333 g) tetraethyl orthosilicate (TEOS) as the Si source. After calcination, the final Ge-UTL product had a Si/Ge ratio of 4.4 (determined by EDX) and a ^29^Si enrichment level of ∼18%.

### Solid-State NMR Spectroscopy

Solid-state NMR spectra were acquired using a Bruker Avance NEO instrument, equipped with a 20 T wide-bore magnet, operating at a Larmor frequency of 168.9 MHz for ^29^Si. Powdered samples of Ge-UTL (∼15 mg, 18% ^29^Si) were combined with ∼15 μL of hydrolyzing solution (either distilled water, 3 or 6 M HCl) inside a PTFE HRMAS insert, which was placed inside a 4 mm ZrO_2_ rotor and rotated at a MAS rate of 5 kHz, using a conventional Bruker HXY probe. ^29^Si chemical shifts are shown in ppm relative to Si­(CH_3_)_4_, using the OSi­(CH_3_)_3_ resonance of octakis­(trimethylsiloxy)­silsequioxane (Q_8_M_8_) (δ = 11.5 ppm) as a secondary reference. Spectra were acquired at 20, 35, and 50 °C. Temperatures were precalibrated using methanol.

For *in situ* experiments, interleaved acquisition of ^29^Si MAS NMR spectra with recycle intervals of 1 s (averaging 128 transients) and 30 s (averaging 16 transients) was performed to acquire the liquid- and solid-state spectra separately over ∼20 h, using 90° (3.2 μs) pulses with a radiofrequency nutation rate of ∼78 kHz. The first ∼5–8 min of the reaction is not accessible owing to the time required to insert and spin the sample, and to tune the probe. See Section S1 of the Supporting Information for more detail on the acquisition and analysis of the NMR spectra.

Experimental NMR data were analyzed using an Avrami–Erofe’ev (JMAK) type kinetic approach,^43^ which has been used extensively to study many different transformations in the solid state.^23,44–46^ The Avrami–Erofe’ev equation is

$$x_{t}=1-\exp(-kt^{n})$$ 1

where **x**_*t*_ is the relative amount of species **x** at time *t*, *k* is the rate constant and *n* (which can vary between 0 and 4) gives information on the dimensionality and nucleation properties of the process.^43^

### Powder XRD Measurements

For *in situ* PXRD experiments 15 mg of Ge-UTL was mixed with 15 μL of either distilled water, 3 or 6 M HCl and packed into a polyimide tube with an inner diameter of 3.2 mm. Measurements were performed on a STOE STADIP instrument using a Mo X-ray tube with a primary beam monochromator (MoK_α1_ = 0.709 Å). Data were acquired at temperatures between 20 and 80 °C using an Oxford Cryosystems Cobra Plus nonliquid nitrogen cryostream, and taken over the 2θ range from 1 to 20° every 5 (70–80 °C) or 10 min (20–50 °C), with total experiment times between 20 and 113 h. The experimental data were fitted using the program TOPAS^47^ to determine the diffraction peak positions and their full width at half-maximum (fwhm).

### Microscopy and Energy Dispersive X-ray Spectroscopy (EDS)

Scanning electron microscopy (SEM) images were collected using a JEOL JSM-IT200 microscope fitted with a tungsten filament and a secondary electron detector. Samples were attached to a SEM stub using C tape and coated in Au using a Quorum Q150R Au/C coater. Scanning transmission electron microscopy (STEM) was performed on an FEI Titan Themis operating at a 200 kV accelerating voltage. Samples were prepared by dispersing the powders in ethanol and drop casting onto copper-mesh TEM grids with a lacey-carbon support film. Samples were plasma cleaned for 1.5 min in pure Ar immediately before imaging using a Henniker HPT-100 plasma cleaner. A dwell time of 2 ms was used for imaging and care was taken to limit beam exposure of the sample to minimize any beam damage to the specimen. The STEM was equipped with a SuperX-G1 windowless EDS detector for fast EDS mapping. Images were taken before and after mapping for the EDS regions to verify that no beam damage had occurred during acquisition.

## Results and Discussion

The ADOR reaction of Ge-UTL (see Figure 1) involves the controlled disassembly (hydrolysis) of the parent zeolite by selective removal of Ge. Initial hydrolysis under aqueous conditions produces a short-lived, disordered layered intermediate, IPC-2P*, which still contains low levels of Ge. This can be converted through continuing hydrolysis to IPC-1P, a layered Ge-free structure from which all the d4r have been removed. Depending on the conditions used, some silicon species may reintercalate to form the partially connected IPC-2P intermediate. However, an induction period may be required, during which there is little change in the long-range order in the material, before this organization occurs,^23,24^ with the induction time becoming shorter as the temperature of the reaction increases. A fully condensed zeolite, IPC-2, where the silicate zeolite layers are joined by s4r can be formed from IPC-2P after a high-temperature reassembly step.^20–25^ The ^29^Si MAS NMR spectra and PXRD patterns of the Ge-UTL starting material and typical IPC-1P and IPC-2P intermediates are shown in Figures S1.1 and S2.1 of the Supporting Information. Table 1 shows expected values of the *d*_200_ spacing and *Q* ^4^/*Q* ^3^ ratio for idealized, defect-free Ge-UTL, IPC-1P and IPC-2P structures. Note that the Ge-UTL samples used in these reactions had a *Q* ^4^/*Q* ^3^ ratio (as determined using ^29^Si NMR spectroscopy) of between 10.1 and 10.6, suggesting a defect level of ∼9 in every 100 Si atoms, although the overlap of signals (from the presence of multiple T sites and the disorder) makes this value difficult to determine accurately.

**Table 1:** Expected Si *Q* ^4^/*Q* ^3^ Ratios and *d*_200_ Spacings for Idealized, Defect-Free Ge-UTL, IPC-1P, and IPC-2P Materials^20–25^

material	*Q* ^4^/*Q* ^3^	*d*_200_/Å
Ge-UTL	∞	14.4
IPC-1P	2.75	10.5
IPC-2P	7.00	11.7

### *In Situ* Experiments

The hydrolysis of ^29^Si-enriched Ge-UTL was monitored using *in situ* NMR spectroscopy at three different temperatures (20, 35, and 50 °C) in each case using distilled water, 3 and 6 M HCl as the hydrolyzing solution. In the original CLASSIC^38^ NMR experiment, cross-polarization (CP^48^), was used to selectively observe the solid-state signals in the NMR spectrum, exploiting the dipolar coupling between two nuclei that is suppressed by rapid motion in solution. For the present study, the inherently nonquantitative nature of CP (which depends on internuclear distances) is a problem as quantitative measurements of the proportion of *Q^n^* Si species present are required. In this work, therefore, discrimination between species in the liquid and solid states was attempted by exploiting the expected differences in relaxation rates, with interleaved acquisition of ^29^Si NMR spectra acquired with shorter and longer recycle intervals (see Section S1 of the Supporting Information). This approach has been used successfully in previous work to study intercalation into zeolites,^41^ and the crystallization^49^ and guest exchange^42^ processes of organic inclusion compounds.

Figure 2 shows two examples of the variation in the ^29^Si MAS NMR spectra as a function of time during the *in situ* reaction of Ge-UTL with H_2_O at 20 °C and 6 M HCl at 50 °C. The complete set of *in situ* spectra is available in Figures S1.2 and S1.3 of the Supporting Information. The spectra show two broad signals corresponding to *Q* ^4^ (centered at approximately −113 ppm) and *Q* ^3^ (centered at approximately −101 ppm) Si species in the solid. There is no evidence for significant amounts (i.e., at levels above the noise) of *Q* ^2^ or *Q* ^1^ species. This is to be expected as *Q* ^2^ and *Q* ^1^ species are very sensitive to hydrolysis and tend to be short-lived, at least compared to the time required to collect the NMR spectra. The complex lineshapes and large line widths reflect both the high number of crystallographically distinct Si species in the UTL structure (12) and the disordered nature of the zeolites upon disassembly. In each of the cases shown, and indeed in all of the reactions carried out, no signals are seen that could be attributed to Si species in solution. These signals would be expected to be considerably narrower owing to rapid tumbling and should be observed preferentially in the spectra acquired with the shorter recycle interval (as observed in previous work on the intercalation of TEOS into IPC-1P^41^). However, no such signals are apparent, and there is little difference between the spectra acquired with the 1 and 30 s recycle intervals (see Figures S1.2 and S1.3). This suggests that under these conditions (i.e., at the pH and temperature shown and at these reaction volumes), the Si released during the disassembly step is not present in any significant amount in solution but likely remains trapped between the layers in multiple different environments or, depending on the conditions, reconnects quickly to the zeolitic framework. Plots of the variation of the ^29^Si *Q* ^4^/*Q* ^3^ ratio as a function of time are shown for all of the reaction conditions in Figure 3 and demonstrate that the progress of the reaction varies both with the temperature and the level of acidity (as discussed below).

**Figure 2:**
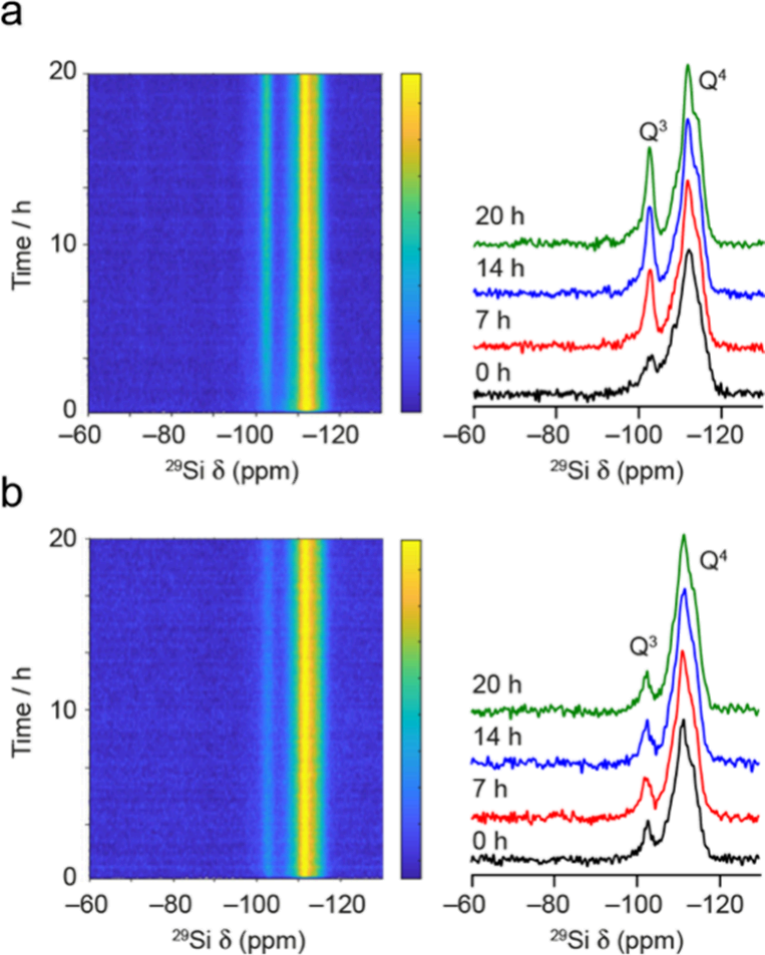
^29^Si (20.0 T, 5 kHz) MAS NMR spectra (shown as intensity contour plots) acquired during the *in situ* reaction of Ge-UTL with a hydrolyzing solution of (a) H_2_O at 20 °C and (b) 6 M HCl at 50 °C, with a recycle interval of 30 s. In each case, spectra acquired after 0, 7, 14, and 20 h of reaction are also shown.

**Figure 3:**
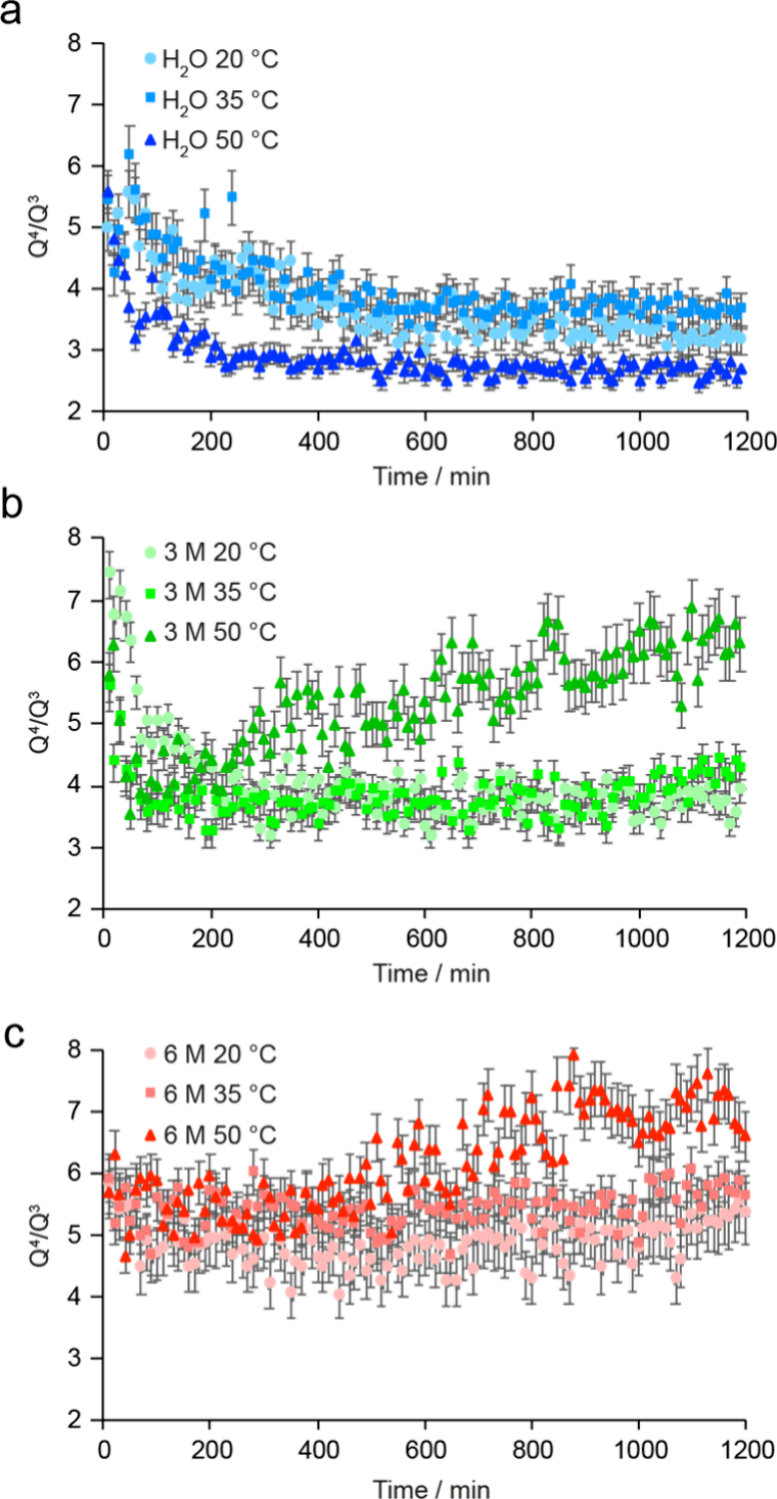
Plots showing the variation in the *Q* ^4^/*Q* ^3^ intensity ratio extracted from the ^29^Si (20.0 T, 5 kHz) MAS NMR spectra acquired (with a recycle interval of 30 s) during the *in situ* reaction of Ge-UTL with (a) water (blue points), (b) 3 M HCl (green points), and (c) 6 M HCl (red points). Data from reactions carried out at different temperatures are denoted by circles (20 °C), squares (35 °C), and triangles (50 °C).

Figure 4 shows two examples of the PXRD patterns (shown as intensity contour plots) collected during the *in situ* reaction of Ge-UTL with H_2_O at 50 °C and 6 M HCl at 50 °C. The complete set of *in situ* PXRD patterns is available in Figures S2.2 and S2.3 of the Supporting Information. The intense peak between 2.7° and 3.5° 2θ shown expanded in the extracted patterns arises from the {200} planes and can be related to the (average) spacing of the zeolitic layers. This spacing is shown plotted as a function of time for all *in situ* XRD reaction conditions in Figure 5. In all cases, the *d*_200_ spacing decreases from the value seen in Ge-UTL (∼14.4 Å) as the parent zeolite is disassembled and the d4rs between the layers are removed (as shown in Figure 1). Although not all reactions were completed within the time studied, a consistent *d*_200_ spacing is obtained under some conditions, suggesting the end product has been reached, but these do not match the values expected for idealized IPC-1P and IPC-2P (given in Table 1). This is not unexpected as in a closed, low-volume system such as the one employed here some of the species that have been disconnected from the parent zeolite (e.g., the germanium atoms) can remain trapped between the layers, as seen previously by both X-ray pair distribution function (PDF) analysis^47^ and EXAFS.^10^ These experiments are particularly enlightening because they show the local structural changes around the Ge on hydrolysis; in water the PDF shows that initial attack is by water at Ge, while in HCl solution this initial attack is by chloride (see later). To remove any germanium-containing species trapped between the layers and reach the ideal *d*-spacing requires a flow system, a much larger volume of solution to drive the deintercalation of these trapped species or a postreaction washing step. In this study we are studying the reaction *in situ* and so all the characterization is completed before any final washing step. However, it should be noted that such postreaction washing removes all the Ge-containing material and leaves, assuming the reaction has gone to completion, pure-phase products (either IPC-1P or IPC-2P).

**Figure 4:**
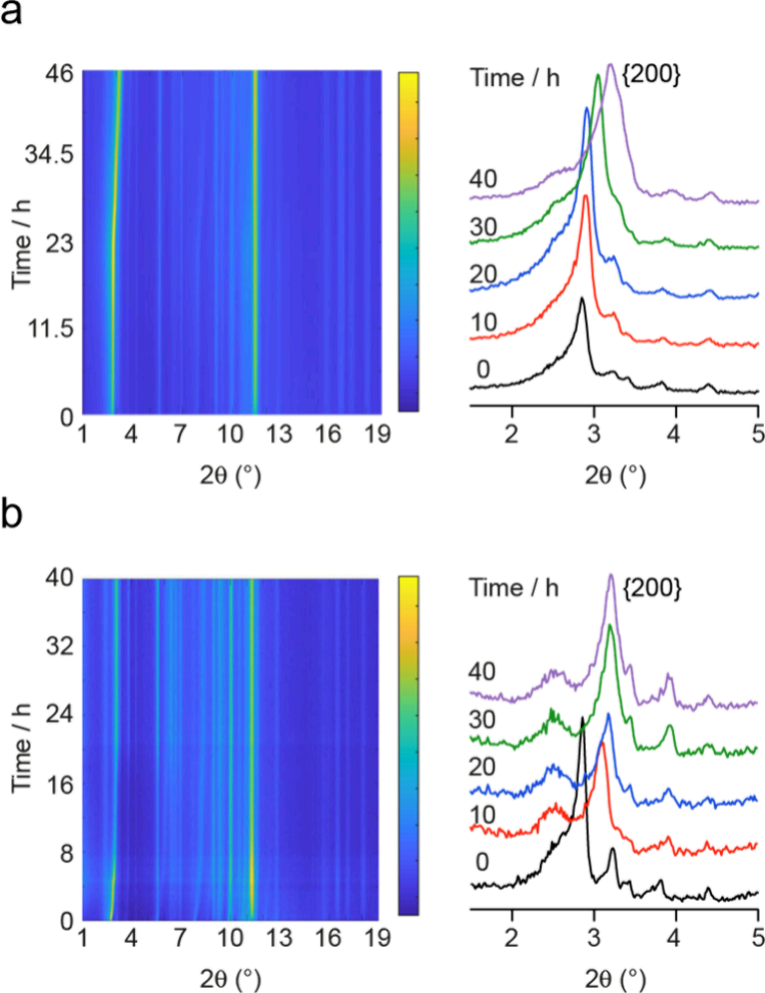
PXRD patterns (shown as intensity contour plots) acquired during the *in situ* reaction of Ge-UTL with a hydrolyzing solution of (a) H_2_O at 50 °C and (b) 6 M HCl at 50 °C. Expansions of the 1.5–5° 2θ range of individual patterns are also shown and overlaid.

**Figure 5:**
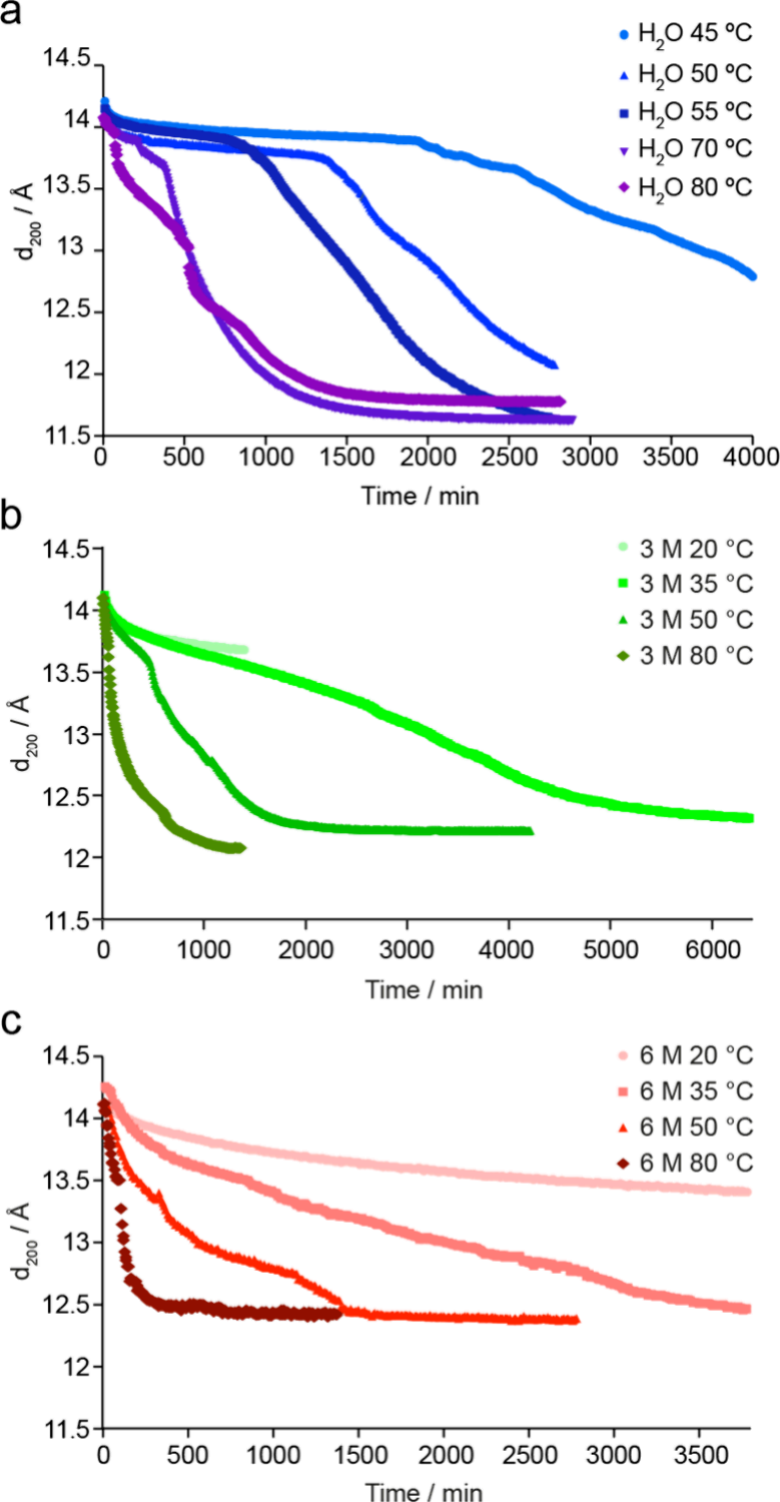
Plots showing the variation in the *d*_200_ spacing extracted from the PXRD patterns acquired during the *in situ* reaction of Ge-UTL with (a) water, (b) 3 M HCl, and (c) 6 M HCl. Data from reactions carried out at different temperatures are denoted by different symbols and colors.

Interestingly, Figure 5 shows that in many cases the *d*_200_ spacing does not vary continuously throughout the reaction but often shows steps or plateaus where a constant value is observed, suggesting a “staged” reaction. This is in notable contrast to the results from the *in situ* NMR experiments where the *Q* ^4^/*Q* ^3^ ratio shows an almost continuous variation of the local structure as a function of time. Note also that in both experiments the *Q* ^4^/*Q* ^3^ ratio (NMR) and *d*_200_ spacing (XRD) does not start from the ideal value for UTL given in Table 1. This is because the hydrolysis reaction is so fast that it starts immediately on adding the water to the system, and so the first 5–8 min of the reaction are missed while the reaction vessel is sealed and loaded into either the NMR spectrometer or the X-ray diffractometer. The exact starting values of the *Q* ^4^/*Q* ^3^ ratio and *d*_200_ spacing vary slightly depending on the exact time taken to load the samples on the instruments.

Examples of the changes in *Q* ^4^/*Q* ^3^ and *d*_200_ spacing taking place in the *in situ* reactions of Ge-UTL with water (at 50 °C for NMR spectroscopy and 70 °C for PXRD) and with 3 M HCl (at 50 °C in both experiments) are shown in Figure 6. In the discussion below, these results are used to explain how the two sets of *in situ* data can be combined to give a much improved picture of the local and long-range structural changes occurring during the ADOR process at different conditions.

**Figure 6:**
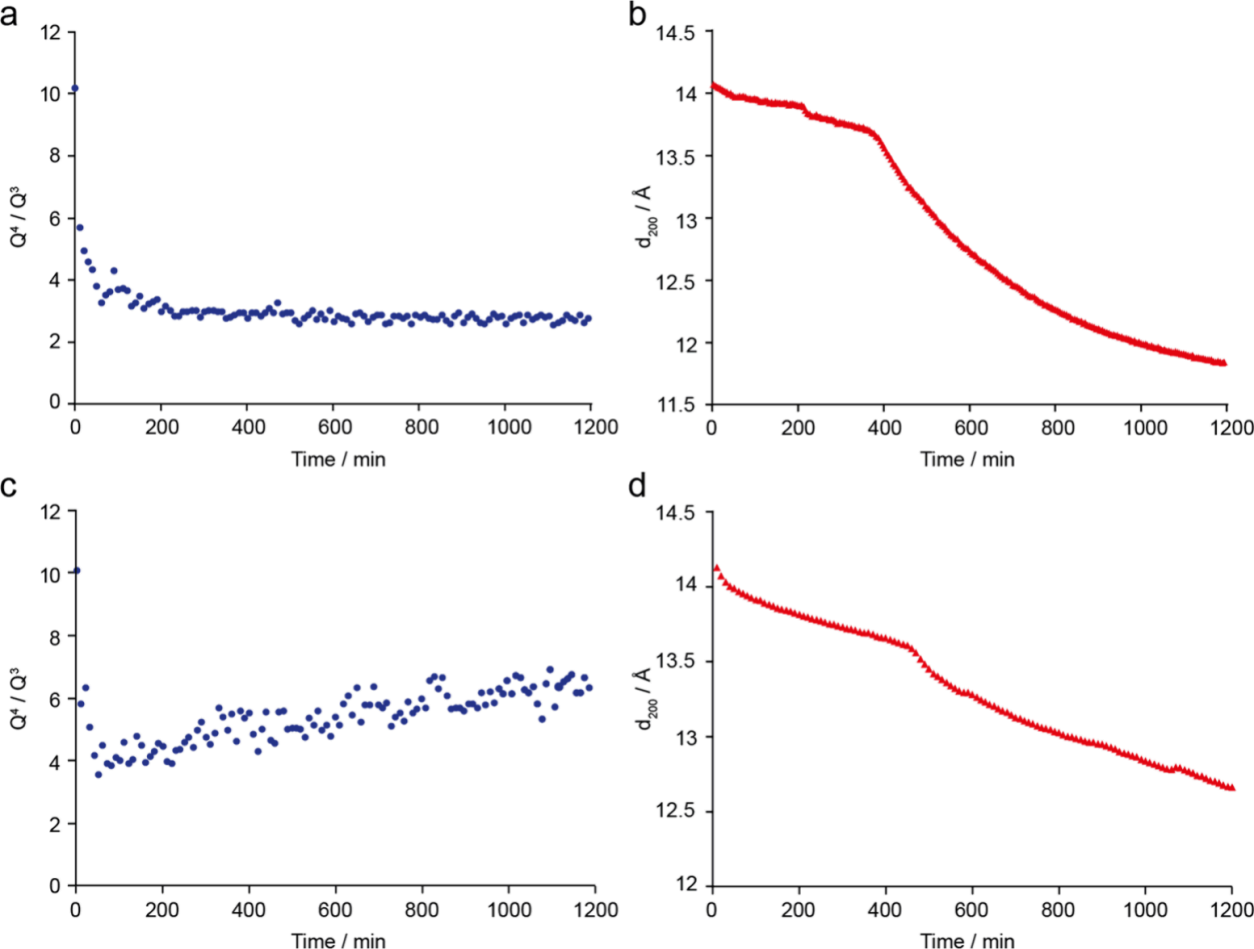
Comparison of data from NMR spectra (blue) and PXRD patterns (red) acquired during the *in situ* reaction of Ge-UTL with (a, b) water and (c, d) 3 M HCl at (a, c, d) 50 °C and (b) 70 °C.

### Reactions with Water and Formation of IPC-1P

In order to gain insight into the mechanism(s) taking place in the ADOR reactions studied, the first step is to consider the hydrolysis reaction that takes place when only water is used. The ADOR disassembly process involves the hydrolysis and removal of the d4r units in Ge-UTL which, if this continues to completion, would lead to the IPC-1P layered intermediate (as in Figure 1). *In situ* NMR spectroscopy (Figure 3a and Figure 6a) shows that the *Q* ^4^/*Q* ^3^ ratio decreases during the reaction of Ge-UTL with water from ∼10.1 in the starting material to ∼6 within the time taken to insert and spin the sample, before decreasing further as the reaction progresses. At 50 °C, an almost consistent ratio (of ∼2.5) is reached by the end of the reaction, suggesting that the IPC-1P layered intermediate has been formed. This would have an idealized *Q* ^4^/*Q* ^3^ ratio of 2.75 (see Table 1), but the *Q* ^3^ defects present in the original Ge-UTL starting materials result in a lower *Q* ^4^/*Q* ^3^ ratio in reality. A similar disassembly process is seen at lower temperatures (Figure 3a), but with a slower rate, and the *Q* ^4^/*Q* ^3^ ratio still decreases (slowly) even after 20 h of reaction. Note that different batches of Ge-UTL starting material were used for the reaction at 35 °C to those at 25 and 50 °C , which may lead to some small differences in reactivity or rate. It is not clear whether the final product obtained at 50 °C would also be reached at longer times in the reactions at lower temperature, or whether there is some limit to the extent of disassembly taking place at lower temperatures. The disassembly behavior seen is similar to that observed by Henkelis et al.^23^ in an *ex situ* study of the hydrolysis of Ge-UTL with water. This reaction initially produced IPC-1P, which after an induction period underwent a subsequent rearrangement to IPC-2P. The length of the induction period varied with temperature, increasing from ∼50 min at 100 °C to 1000 min at 70 °C. No such rearrangement (formally an ADOR “organization” step) is seen in the *in situ* NMR data, but this is not unexpected at the lower temperatures used, which should result in much longer induction periods, if indeed such organization occurs at all on a reasonable time scale close to room temperature. Furthermore, Henkelis et al.^23^ used a large-scale reaction (with 600 mg of Ge-UTL and 120 mL of water), with small aliquots extracted at specific time points. Previous work has shown that Ge-UTL hydrolysis can also be highly dependent on the scale of the reaction, and particularly on the volume of solution used, with the time scales increasing from 5 min (40 mL) to several hours (∼20 μL) in reactions carried out at higher temperatures (90–100 °C).^20,21^

As shown by the example data in Figure 6a, a continuous change in the local structure is observed during the *in situ* NMR experiments. The initial Si/Ge of 4.4 in the parent Ge-UTL material is relatively low, indicating that on average more than half of the T atoms in any d4r are Ge, with the remainder being Si. This means that statistically some d4r will have four Ge atoms and some five. Previous computational work has shown that the materials are most stable when there are Ge–O–Ge bonds in the d4r units.^16^ Figure 7 shows two arrangements of the Ge in a d4r, containing four and five Ge atoms, respectively. It should be noted that, in each case, there are several possible arrangements of the Ge atoms in the d4r, and only one possible arrangement is shown. The reduction in the *Q* ^4^/*Q* ^3^ ratio during the disassembly process is consistent with the process shown in Figure 7, where removal of germanium and silicon atoms from between the layers changes some of the initial *Q* ^4^ silicon species (shown in blue in Figure 7) into *Q* ^3^ species (shown in green). Eventually, the entire d4r is disconnected from the silica-layers to form an IPC-1P like local structure. From the known hydrolysis behavior of germanates and germanosilicates,^3,4,11^ the Ge atoms are expected to be hydrolyzed more easily than the Si species, but as there is a relatively smooth change in the *Q* ^4^/*Q* ^3^ ratio, there appears to be no discrimination between Si and Ge hydrolysis in this experiment. As discussed below, this means it is possible to describe the disassembly process seen using NMR spectroscopy by a single Avrami expression which gives average kinetic parameters for all possible local reactions. Note also that if there are more than four Ge atoms in any one d4r there is the possibility of forming *Q* ^2^ species (shown in yellow in Figure 7). Such species are not visible in the NMR spectra (unlike in previous work on the intercalation of TEOS into IPC-1P^41^), which could indicate that these species are formed only at a low level. However, computational studies have shown that *Q* ^2^ species are less stable than *Q* ^3^ and *Q* ^4^ species and so are also likely to be lost very quickly once they are formed.^51^

**Figure 7:**
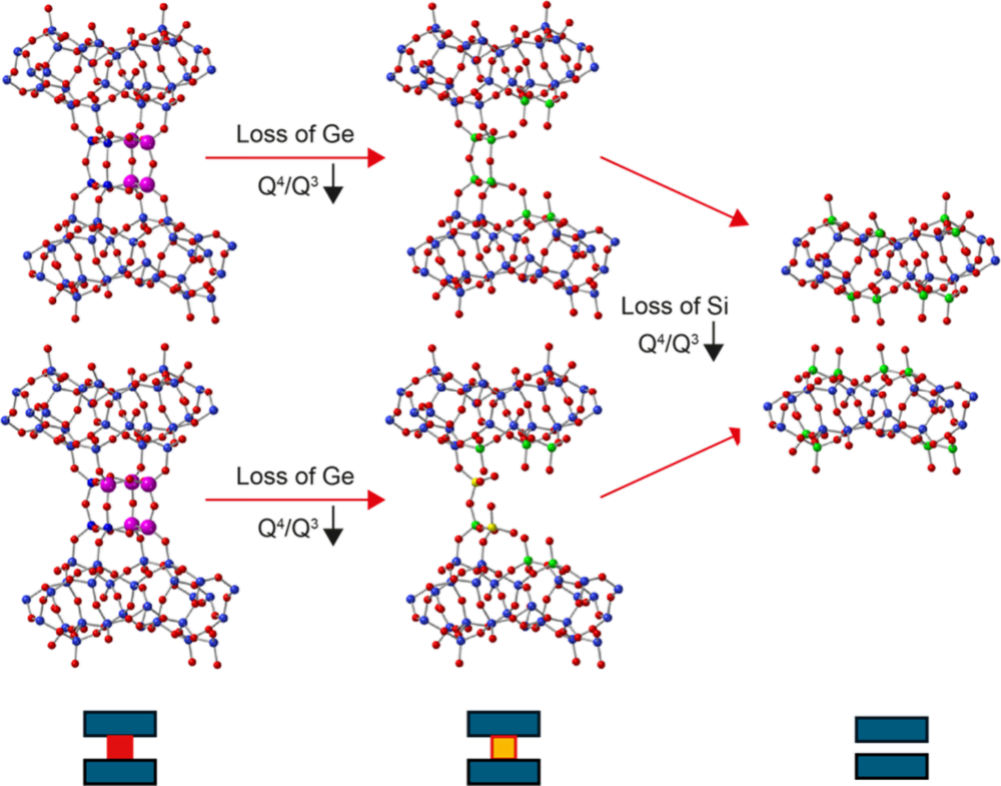
Schematic explaining the changes in the local structure during the reaction of Ge-UTL with water at 70 °C, showing how the loss of Ge and Si from the interlayer space leads to a decrease in *Q* ^4^/*Q* ^3^ as the *Q* ^4^ Si (blue) becomes *Q* ^3^ Si (green) after loss of Ge (pink). Note that the diagram shows initial d4r units with four (top) and five (bottom) Ge atoms, respectively, and that loss of Ge from d4r units that contain only three Si atoms leads to the possible formation of *Q* ^2^ species (yellow). Oxygen atoms are shown in red. Shown at the bottom of the figure are schematic representations of the different local species used in Figure 9.

The kinetics of the *in situ* reaction followed using NMR spectroscopy can be analyzed to gain more quantitative insight into the effects of acidity and temperature, and to help understand the competition between the different steps of the ADOR process. It can be assumed that when the reactions are carried out in water only the disassembly process takes place, leading, for the reaction at 50 °C, to a completely disassembled IPC-1P like product. The change in *Q* ^4^/*Q* ^3^ can be modeled using the Avrami–Erofe’ev equation^43^ as shown in Figure 8. Previous work has suggested that the order of reaction for the disassembly step, *n*_diss_, is likely to be considerably less than 1, reflecting the reduced dimensionality of the reaction. Work by Henkelis et al.^23^ found *n*_diss_ values between 0.2 and 0.4 for large volume *ex situ* hydrolysis of Ge-UTL at higher temperatures (above 70 °C), while recent work on intercalation of TEOS into IPC-1P (a similar, but reversed, process) also found *n*_int_ ≈ 0.4.^41^ Figure 8c shows the best fit to the data from the *in situ* reaction of Ge-UTL with water at 50 °C giving *n*_diss_ = 0.204 and *k*_diss_ = 0.643 min^–0.204^. Although there is some scatter in the data, it is clear from fittings in which *n*_diss_ is fixed at values between 0.1 and 0.5 and *k*_diss_ is varied (see Figure S3.1 in the Supporting Information) that values of *n*_diss_ both below and above 0.2 produce significantly worse fits, giving confidence in the robustness of the result. As shown in Figure 8 (parameters in Table S3.1 of the Supporting Information), similar results are also obtained for the reactions of Ge-UTL with water at 20 and 35 °C, although it should be noted that the reaction does not go to completion in 20 h at these temperatures.

**Figure 8:**
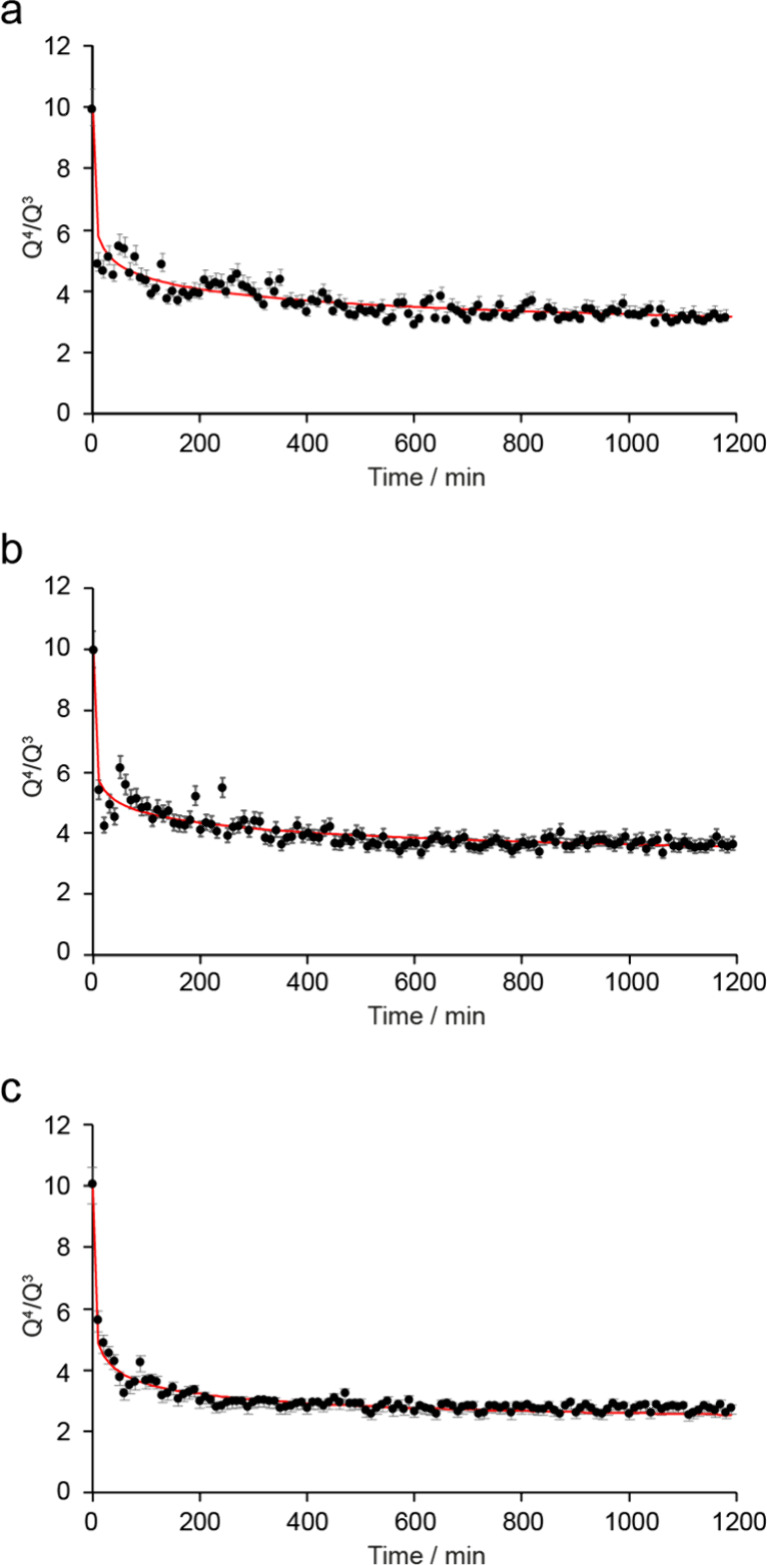
Plots showing the best fit from the kinetic analysis for the experimental data from the *in situ* reaction of Ge-UTL with water at (a) 20 °C, (b) 35 °C, and (c) 50 °C. Corresponding kinetic parameters (*n*_diss_ and *k*_diss_) for the disassembly process are given in Table S3.1 of the Supporting Information.

The *in situ* PXRD experiments (Figure 5a and Figure S2.2) also show an increase in the rate of the ADOR disassembly process using water with increasing temperature. The *d*_200_ spacing decreases from just above 14 Å (for Ge-UTL) as disassembly takes place. However, at temperatures below 55 °C very little change is seen in the *d*_200_ spacing after 20 h, despite a decrease in the *Q* ^4^/*Q* ^3^ ratio being clearly shown by NMR spectroscopy. When the temperature is increased a series of changes in the *d*_200_ spacing are seen. For example, at 70 °C (Figure 6b) rapid changes are seen initially, after ∼200 min and after ∼400 min, but with little variation between these points, suggesting that a stepwise or staged process is taking place. The time a material spends at each stage decreases as temperature increases, as can be seen from the reaction at 80 °C in Figure 5a). Although bulk hydrolysis at the lower temperatures is clearly incomplete, a consistent and similar *d*_200_ spacing is reached for the reactions between 55 and 70 °C, suggesting that the expected IPC-1P like product has been successfully formed. However, the observed *d*_200_ spacing of ∼11.6 Å does not match the value expected for the idealized material (∼10.5 Å) given in Table 1. Very similar PXRD patterns were obtained for the final products of the *in situ* NMR reactions (see Figure S1.5), also with *d*_200_ spacings of ∼11.7 Å (note that these PXRD patterns were acquired a number of days after the NMR experiments were performed – see the Supporting Information). As described above, the fact that the observed *d*_200_ spacings are higher than the idealized value is likely the result of species not fully deintercalating from the material, such that the layers remain further apart than they would be in the fully deintercalated material. In *ex situ* studies samples are often washed, filtered and dried (e.g., at 80 °C for 5 min in the work of Henkelis et al.^23^) before PXRD measurements are taken to confirm the phase(s) formed, which will affect the level and type of material present between the zeolitic layers and therefore their spacing.

The fwhm of the {200} reflection changes as a function of time in a manner that can be broadly understood based on the expected changes in the degree of crystalline order for a process in which the average *d*_200_ spacing changes as the reaction proceeds (see Figure S2.7 in the Supporting Information). During periods of the reaction in which there is no major change in the average *d*_200_ spacing, the fwhm remains relatively constant. However, in periods where the average *d*_200_ spacing changes rapidly, the fwhm also changes, but in a manner that depends on how far the reaction has gone toward completion. For example, for the reaction with water at 55 °C (Figure S2.7c) there is no major change in fwhm until after the inflection point at just below 1000 min, which corresponds to a rapid change in the average *d*_200_ spacing. After this time, the fwhm increases as the average *d*_200_ spacing decreases, as each contraction of interlayer distances reduces the overall crystalline order of the crystallites, resulting in peak broadening. However, as the process nears completion, a point is reached at which additional layer contractions actually lead to an increase in crystalline order, as the final product of the reaction is an ordered or pseudo-ordered phase, in this case IPC-1P. As a result, the fwhm reaches a maximum value at ∼2000 min for the reaction at 55 °C and then decreases again until the reaction is over and the fwhm then remains constant with time. All reactions with water show broadly the same behavior in the variation of fwhm as a function of time, although under the different conditions studied these changes occur at different rates and at different times during the process.

Figure 9 shows a schematic of how the disassembly process that occurs during the reaction of Ge-UTL with water at 70 °C could correspond to the stepwise changes in the *d*_200_ spacing seen in the XRD experiments (e.g., in Figure 6b). At the very early stages of reaction there is likely to be rapid but random hydrolysis of the d4r units removing Ge to leave partial d4r units between the layers (shown in yellow) but resulting in no significant change to the layer spacing. The Si in the d4r is then lost more slowly. As this process proceeds, there comes a point when sufficient hydrolysis has occurred between two layers that they can move closer together to form the contracted IPC-1P like motif. This is the structural feature that is probed by the position of the {200} reflections in the PXRD. The need for all the sites between a pair of layers to have completely hydrolyzed necessarily means that changes in the long-range structure probed by XRD can lag significantly behind the local changes probed by NMR spectroscopy. However, the plateaus in the *d* spacing seen in the XRD data suggest that this process cannot be random and must proceed in a staged manner, similar to that seen in many other reactions of layered materials (such as intercalation into graphite^52–54^ ). For the reaction of Ge-UTL with water at 70 °C (Figure 6b) there are two plateaus in the graph that shows the change in the average *d*_200_ spacing with time: one tending to ∼13.9 Å and one tending to ∼13.6 Å. These plateaus (denoted stage 1 and stage 2 respectively) correspond to ∼20% and ∼30% of the layers having moved closer together to form the contracted structure. These values have been estimated by considering the starting *d*_200_ value for Ge-UTL, where all layers are separated by d4r units, and the value of *d*_200_ at the end of the reaction, where we assume that all the interlayer distances have moved to their final value, and then computing the percentage of layer spacings that have contracted based on the average *d*_200_ spacing observed from the PXRD data. Clearly, there will be considerable uncertainty in the percentage of contracted layers estimated in this way because of the possibility that species may be trapped between the layers in the experimental system.

**Figure 9:**
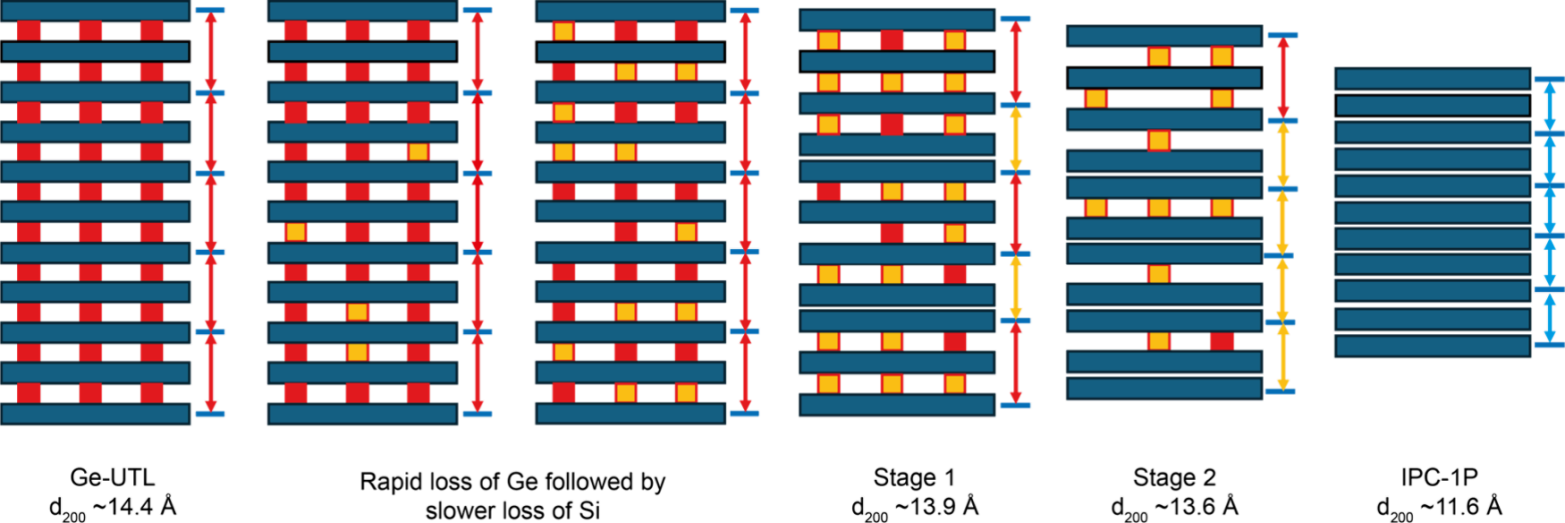
Schematic representation of the reaction of Ge-UTL with water at 50 °C showing how the observed *d*_200_ spacing varies with time (from *t* = 0 at the left of the diagram). The color coding represents the different local structures shown in Figure 7, with red and yellow squares representing d4r units before and after loss of germanium, respectively, and the blue rectangles representing the silicate layers. Interlayer spacings of different sizes are shown by the different colored arrows.

Figure 9 shows two possible schematic structures for stages 1 and 2 of the reaction. If the contractions between layers were completely random and all pairs of layers were equally likely to contract, there would be a smooth change in the average *d*_200_ spacing during the whole reaction. However, since we have clear evidence of staging in the process this cannot be the case. As one pair of layers comes closer together this must affect the likelihood that other pairs of layers will contract. This is not unusual in staged intercalation and deintercalation reactions, such as those of graphite.^52–54^ PXRD provides the average *d*_200_ spacing, not the relative positions of the contracted pairs of layers. Two extreme possibilities could be suggested in which (a) the most favorable contractions occur if there are no previously formed IPC-1P layers close by, and (b) the probability of contractions increases close to any previously formed IPC-1P layers. In case (b), the limit would be the continual growth of an IPC-1P-like region which expands through the crystal as the reaction progresses. As with the random contraction of layers this would lead smooth changes in the average *d*_200_ spacing. The staged nature of the reaction observed in our results suggests that case (a) is more likely, i.e., that contraction of layers occurs only if there are no previously formed IPC-1P layers close by. The average *d*_200_ spacings observed suggest that at stage 1 of the reaction there are no IPC-1P layers within two layers of those that are undergoing the contraction (i.e., a contracted layer cannot have another contracted layer as its nearest or nearest but one layer). Once this constraint has been satisfied by as many layers as possible, the next most favorable situation is to have at least one uncontracted layer between contracted layers (i.e., stage 2). Again, once this constraint has been satisfied throughout the structure, the next most favored process is to contract all the remaining layers to form the final IPC-1P like material. It should be noted that this mechanism does not necessarily result in an ordered material at stage 1 or stage 2, and no such order is seen in the PXRD patterns. As shown in Figure 5a, as the temperature is increased and the rate of the reaction increases, it becomes more difficult to discern the stages of the reaction, indicating that any constraints in the order of layer contractions can be overcome by increasing the temperature.

The staged behavior described above helps to explain the previously reported and unusual structure of IPC-6, a final reassembled zeolite where TEM shows that the material has a similar structure to the stage 2 material described here.^20^ It would, of course, be desirable to follow the reaction using *in situ* TEM to directly observe the stages of the reactions. Unfortunately, the intermediate materials are very susceptible to connecting in the electron beam, i.e., initiation of the reassembly step in the ADOR process. It is not possible, therefore, to directly image any of the staged intermediates with enough resolution to show any detailed structural features. However, we should note that the final reassembled materials (IPC-4 and IPC-2) can be studied using electron microscopy, and their structures have even been solved by electron crystallography.^55^

### SEM and TEM Images

Taken together the evidence above suggests that the *in situ* hydrolysis of Ge-UTL produces an IPC-1P like material, which has a local structure similar to IPC-1P but with the zeolitic layers more widely spaced than would be seen in the ideal material. Figure 10a shows SEM images of the initial Ge-UTL and of the product of the *in situ* reaction of Ge-UTL and water at 50 °C. Ge-UTL contains elongated stacks of crystallites, and this sample morphology persists after the reaction with water with no significant differences except for small changes to the surface roughness and the formation of small particles at the crystallite surfaces. TEM images of the same samples are also shown at two different magnifications in Figure 10a. These confirm the presence of small cube-like crystals on the surface of the stacked zeolite crystals after reaction with water, along with some larger agglomerates. EDS measurements of the initial Ge-UTL (Figure 11a) and after the *in situ* reaction with water at 70 °C (Figure 11b) provide insight into the nature of the materials present. A homogeneous distribution of Si and Ge is seen for Ge-UTL, whereas there is a clear distinction between the zeolitic crystals (which are essentially Ge-free) and the small cube-like crystals which contain only Ge and no Si, suggesting their identity as GeO_2_, which can be confirmed using PXRD (as shown in Figure S2.9 in the Supporting Information). GeO_2_ was also seen (by both PXRD and ^17^O NMR spectroscopy) in previous work studying the mechanochemical reaction of Ge-UTL with water, where its presence was attributed to the low volume of solvent present.^56^ Figures 10 and 11 suggest that little Ge remains in the interlayer spaces in the zeolite at the end of the *in situ* reaction, but that the larger *d*_200_ spacing seen in the IPC-1P like products is likely to result from the presence of intercalated Si species.

**Figure 10:**
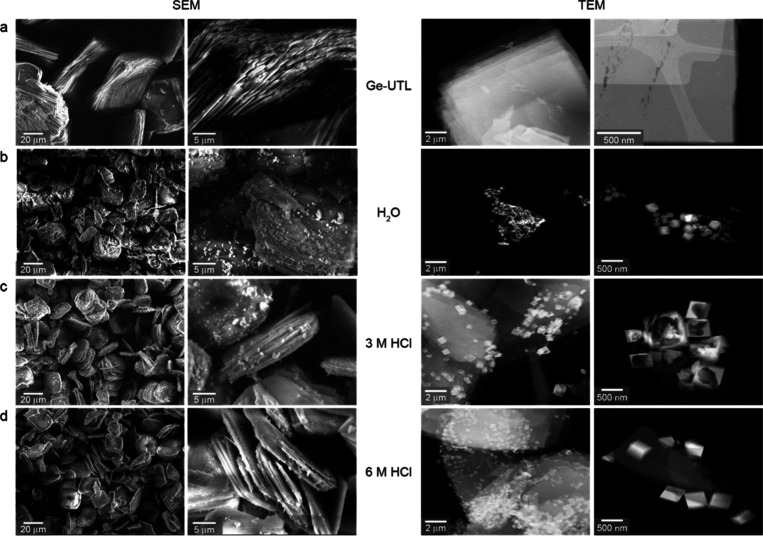
SEM (left) and TEM (right) images of (a) Ge-UTL and the products of the reactions of Ge-UTL with (b) H_2_O at 50 °C (SEM) and 70 °C (TEM), (c) 3 M HCl at 50 °C, and (d) 6 M HCl at 50 °C monitored using *in situ* PXRD at magnifications of ×750 and ×3500 (SEM) and ×5000 and ×14,000 (TEM).

**Figure 11:**
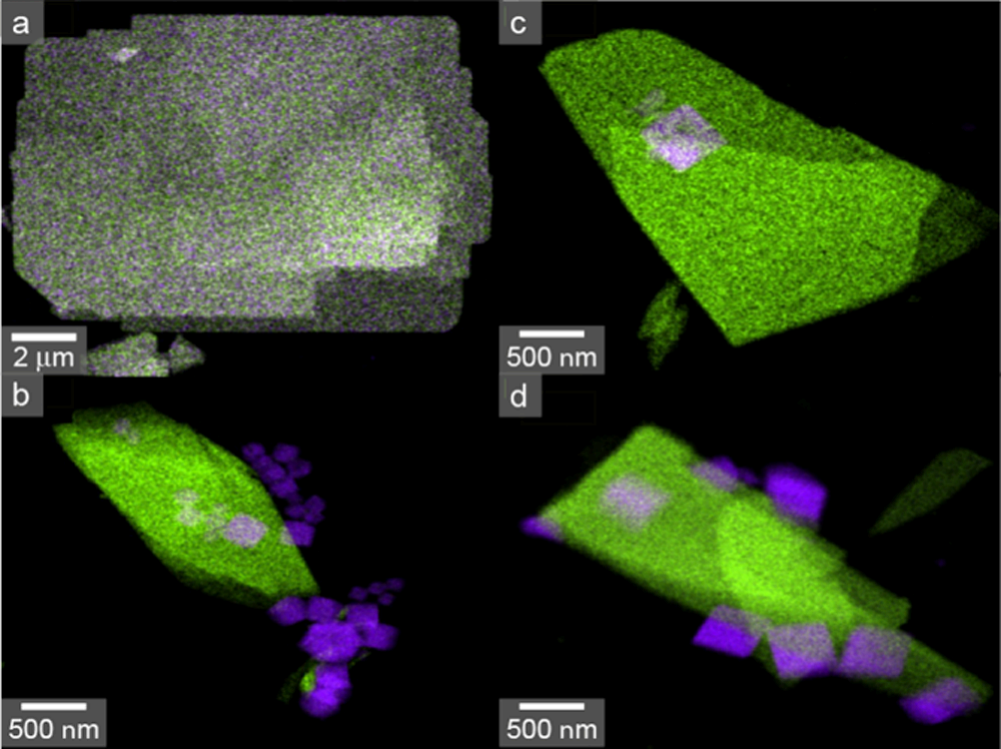
EDS elemental maps of (a) Ge-UTL and the products of the reaction of Ge-UTL with (b) H_2_O at 70 °C, (c) 3 M HCl at 50 °C, and (d) 6 M HCl at 50 °C monitored using *in situ* PXRD, showing the distribution of Si (green) and Ge (purple).

### Reactions with Acidic Solution and Formation of IPC-2P

A range of previous Ge-UTL ADOR studies have shown that acidic hydrolysis changes both the rate of the reactions and the processes that take place leading to different products. For example, work by Wheatley et al.^57^ on *ex situ* reactions involving 1 g of zeolite and 250 mL of solution at 95 °C revealed that low acidity favored deintercalation of Si and the formation of IPC-1P (and subsequently IPC-4 after reassembly), whereas higher acidity favored an interlayer silicon rearrangement and the formation of IPC-2P (and subsequently IPC-2). The authors showed that the *d*_200_ spacing of the final sample (after calcination) was linearly dependent on the acid concentration between 0.1 and 3 M. Similar results were seen by Morris et al.^20^ for Ge-UTL hydrolysis of 250 mg of zeolite in 40 mL of solution at 95 °C, with low acidity promoting the formation of IPC-1P (even at the high temperature) and high acidity leading eventually to IPC-2P but through an IPC-6P intermediate where condensation of layers occurs non randomly (see above).

Figure 3b,c plots the variation of *Q* ^4^/*Q* ^3^ in the ^29^Si NMR spectrum as a function of the duration of the *in situ* hydrolyses carried out under acidic conditions in the NMR rotor. The form of the curves is significantly different to that seen for the reaction with water, with a decrease in *Q* ^4^/*Q* ^3^ in the first instance, before this reaches a minimum value and subsequently increases until the end of the reaction. Figure 12 explains how the development of the local structure during the reaction of Ge-UTL with 3 M HCl at 50 °C leads to the variation of *Q* ^4^/*Q* ^3^ seen in Figure 6c. As in the reaction with water the first step is the loss of Ge from the d4r units. However, in contrast to these reactions, *in situ* PDF experiments have shown that in HCl this occurs by attack of chloride ions at Ge.^50^ This results in a similar decrease in *Q* ^4^/*Q* ^3^ to that seen for reactions with water (albeit at a different rate). Subsequently, however, the remaining Si does not deintercalate but instead rearranges to form structures that connect the layers together, increasing *Q* ^4^/*Q* ^3^. Instead of there being only one disassembly process, as is the case for the reaction of Ge-UTL with water, there are now two competing processes, a disassembly and a rearrangement (formally an organization step). Further evidence for the connection of the layers after rearrangement comes from the fact that the final material (IPC-2P) cannot be chemically swollen by intercalation, while IPC-1P is easily swollen.^58^

**Figure 12:**
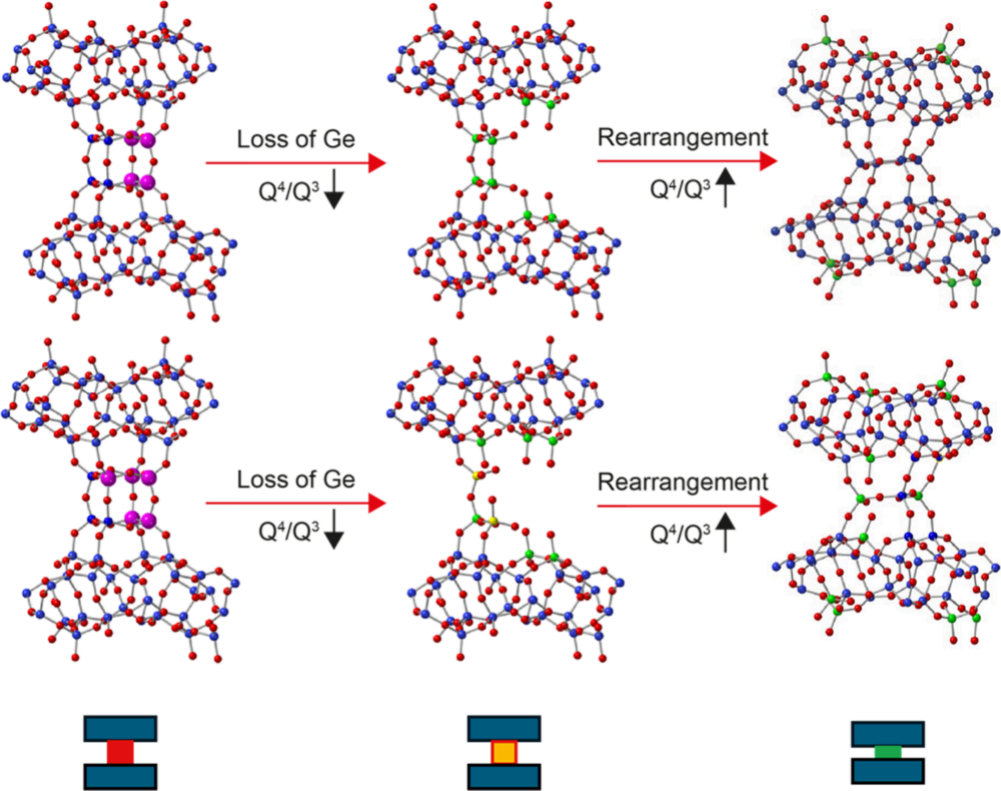
Schematic showing the changes in the local structure occurring during the reaction of Ge-UTL with 3 M HCl at 50 °C, showing how loss of Ge in the interlayer space leads to a decrease in the *Q* ^4^/*Q* ^3^ as *Q* ^4^ Si (blue) becomes *Q* ^3^ Si (green) after loss of Ge (pink). Note that the diagram shows initial d4r units with four (top) and five (bottom) Ge atoms, respectively, and that loss of Ge from d4r units that contain only three Si atoms leads to the possible formation of *Q* ^2^ species (yellow). Subsequent rearrangement of the interlayer silicon species leads to an increase in *Q* ^4^/*Q* ^3^. Oxygen atoms are shown in red. Shown at the bottom of the figure are schematic representations of the different local species used in Figure 14.

An increase in the acid concentration promotes the rearrangement process, and IPC-1P is then never fully formed before this step begins to dominate, and so strictly the IPC-2P never forms a truly “layered” intermediate. This can be seen more clearly in Figure S1.4 of the Supporting Information, where data for reactions at different acid concentrations but the same reaction temperature are compared directly. It is clear from the reactions with 3 M HCl solution (Figure 3b) that the rate of rearrangement also increases with an increase in temperature, and at 50 °C *Q* ^4^/*Q* ^3^ is very close to the value (∼7) expected for an ideal IPC-2P material, confirming the local structure of the product formed.

Unlike the case for the reaction of Ge-UTL with water, where the change in *Q* ^4^/*Q* ^3^ with time can be fitted using one Avrami function, for acid hydrolyses two functions are clearly required, one to describe the decrease in *Q* ^4^/*Q* ^3^ from the hydrolytic disassembly and loss of Ge/Si and one for the acid-induced rearrangement of Si that constitutes the ADOR organization step. One option for fitting the data acquired from the *in situ* NMR experiments under acidic conditions is to fix *n*_diss_ and *k*_diss_ at the values obtained for the disassembly reaction carried out in water at the same temperature, with only the parameters for the organization step *n*_org_ and *k*_org_ then allowed to vary. However, as shown in Figure S3.3 in the Supporting Information, for the reactions carried out at 50 °C with 3 and 6 M HCl, this gives a very poor fit, confirming that the disassembly process itself is different in the presence of acid (with higher *n*_diss_ as well as a faster rate). As described above, this is in good agreement with previous *in situ* PDF work which confirmed that chloride ions are involved in the disassembly process in the presence of HCl.^50,59^ It was not possible to vary all four kinetic parameters in the fitting process, as it was found that the best fit solutions depended significantly on the initial conditions used, resulting in unphysical values of the parameters. For the reactions carried out with 3 and 6 M HCl, Figure 13 shows the best fits obtained with *n*_diss_ and *n*_org_ fixed at 0.4 (in agreement with results from previous work^23,41^ ) and with *k*_diss_ and *k*_org_ varied. Corresponding parameters are given in Table S3.2 of the Supporting Information. It is clear that the data from all reactions can be fitted well by two competing processes; a fast disassembly process and a slower organization step (see Table S3.2). The point at which the organization process begins to be the more dominant contributor to the changes in *Q* ^4^/*Q* ^3^ (i.e., the point in each plot in Figure 13 where the green line crosses the blue line), is plotted in Figure S3.4a. The minimum *Q* ^4^/*Q* ^3^ value seen is ∼3.5–4 for the reactions in 3 M HCl (see Figure S3.4b), which increases to 4.5–5 for reactions at higher acidity, consistent with the organization step becoming dominant earlier in the reaction, reflecting that this is determined by the increased rate of rearrangement at the higher acid concentration. The final *Q* ^4^/*Q* ^3^ is close to 4 for the reactions in 3 M HCl at 20 and 35 °C but increases significantly to ∼6.5 when the temperature is raised to 50 °C, showing the importance of temperature in driving the organization (see Figure S3.4b). A similar observation can be made for reactions in 6 M HCl, but with higher ratios in each case ( ∼5–5.5 at lower temperature and ∼7 at 50 °C) confirming an IPC-2P like product.

**Figure 13:**
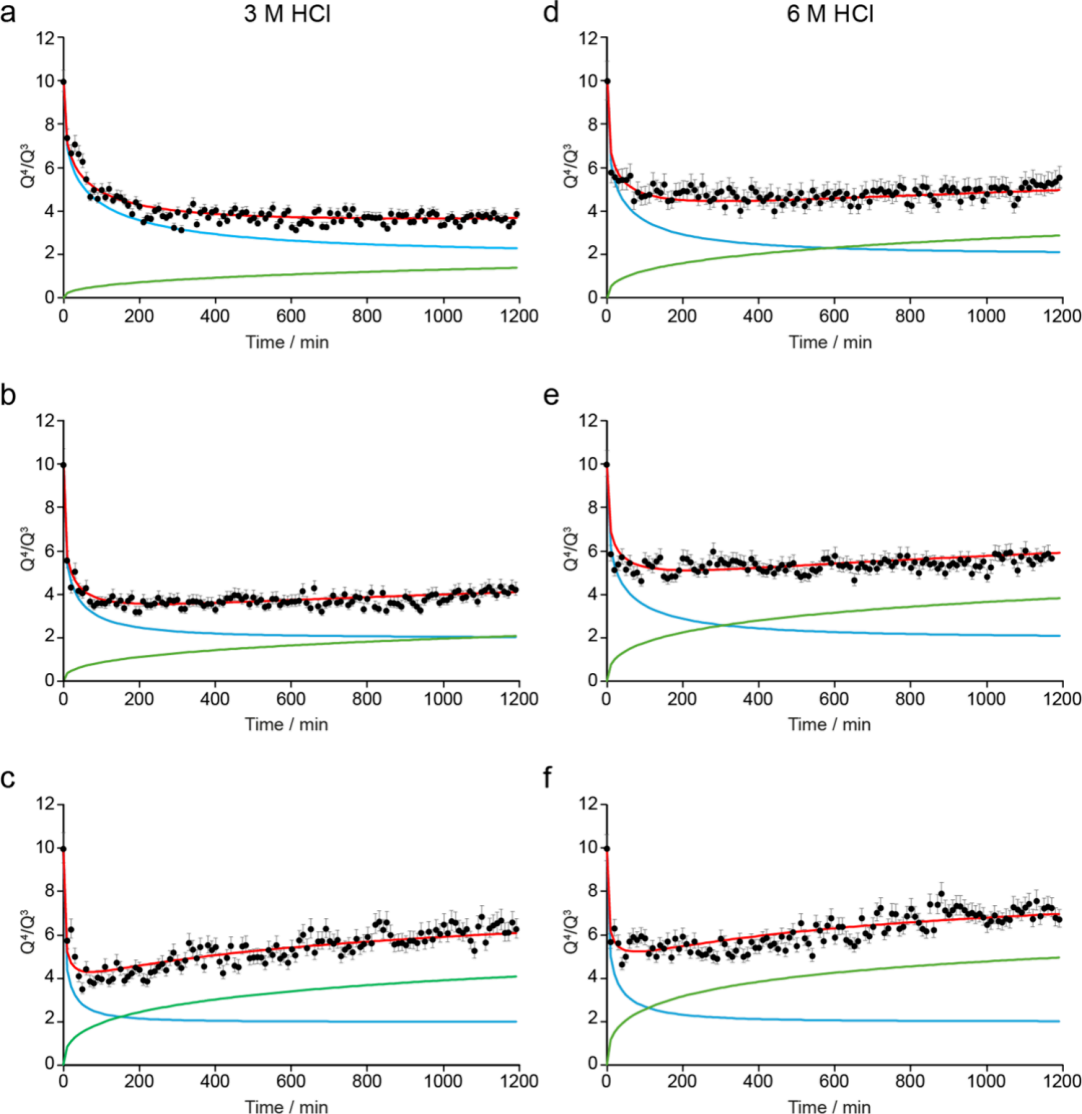
Plots showing the best fit from the kinetic analysis for the experimental data from the *in situ* reaction of Ge-UTL with (a–c) 3 M HCl and (d–f) 6 M HCl at (a, d) 20 °C, (b, e) 35 °C, and (c, f) 50 °C, fixing both *n*_diss_ and *n*_org_ at 0.4 and varying *k*_diss_ and *k*_org_. Kinetic parameters are given in Table S3.2 of the Supporting Information.

The *in situ* PXRD experiments (Figure 5 and Figures S2.3, S2.5, and S2.7) also show evidence for a more complex reaction taking place, with changes in the position and fwhm of the {200} reflection seen at earlier reaction times with increasing temperature. The *in situ* XRD data for the reaction of Ge-UTL with HCl at 50 °C (Figure 6d) shows a similar staging behavior to that described above, except that only one very clear plateau is visible (and so only one major identifiable stage is present) at a *d*_200_ spacing of around 13.5 Å. This must be caused by full rearrangement of one pair of silica-rich layers, reducing the likelihood of rearrangement occurring in adjacent or close zeolitic layers. Increasing both the temperature and the acidity makes the staging less easy to resolve (as seen in Figure 5b,c). A possible overall mechanism for the changes to the average structure seen by XRD is shown in Figure 14.

**Figure 14:**
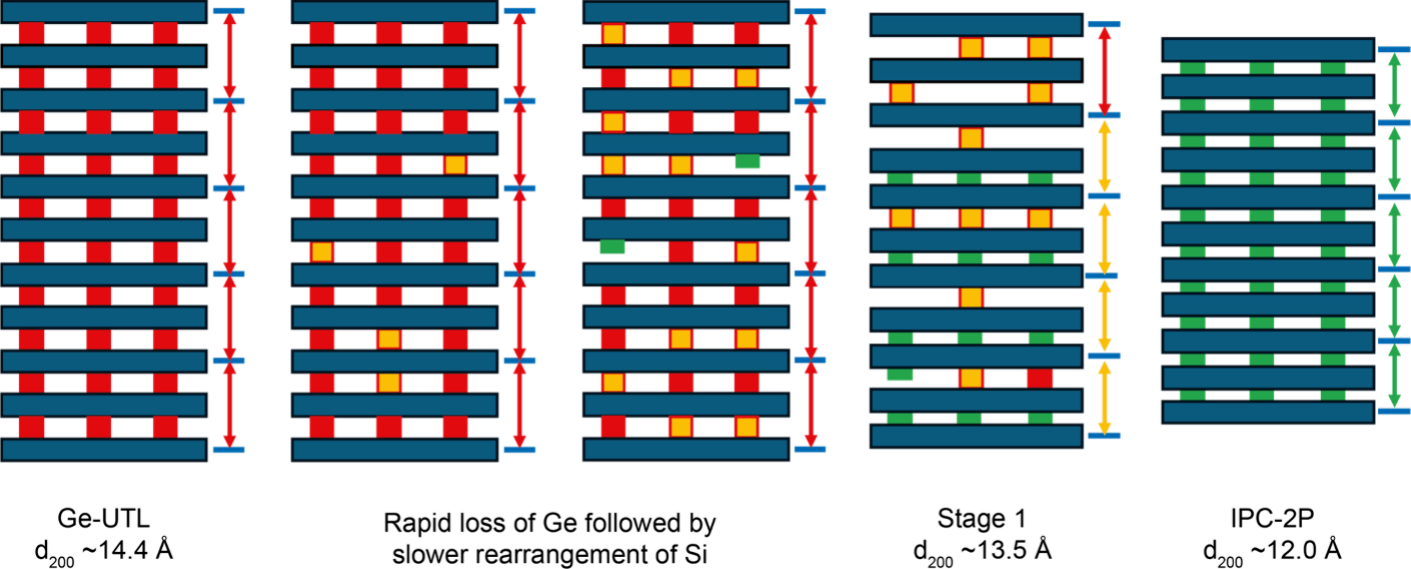
Schematic representation of the reaction of Ge-UTL with HCl at 50 °C (Figure 6d) showing how the observed *d*_200_ spacing varies with time (from *t* = 0 at the left of the diagram). The color coding represents the different local structures shown in Figure 12, with red and yellow squares representing d4r units before and after loss of Ge, respectively, the green rectangles representing s4r of Si after rearrangement, and the blue rectangles representing the silicate-rich zeolitic layers. Interlayer *d* spacings of different sizes are shown by the different colored arrows.

Further evidence for the formation of an IPC-2P like final phase in many of the *in situ* PXRD reactions is again provided by microscopy (acquired for the material recovered at the end of the experiment) in Figure 10c,d. The SEM images show that the sample morphology of the products of the acidic hydrolyses at 50 °C are similar to those seen for hydrolyses in water, with elongated stacks of crystallites present, although these now appear to have smaller particles on their surfaces. TEM clearly shows that the larger zeolite crystals are covered in smaller cubes, more of which appear hollow at the lower acid concentration, which can be confirmed as GeO_2_ by EDS (Figure 11c,d), produced by hydrolysis of Ge chloride species.

At higher temperatures and at the highest levels of acidity there is evidence of a third possible reaction beginning to occur– the breakdown of the silicate structure itself, which leads to extra peaks in the PXRD patterns (see Figure S2.5), with the resultant, ordered SiO_2_ visible in high-resolution TEM as a small number of ∼500 nm triangular particles, which can be confirmed as SiO_2_ using EDS (see Figure S2.9). This information places a limit on the conditions of temperature and acidity for which the ADOR process will be successful.

## Conclusions

The combined application of *in situ* NMR spectroscopy and *in situ PXRD* studies have allowed the correlation of local changes in structure with longer range changes in layer stacking in the ADOR-based manipulation of zeolite structures. A combination of structural and kinetic analysis allows mechanisms to be proposed for the reactions under several different conditions which are consistent with both the *in situ* NMR and PXRD studies. Several new features of the reaction have been discovered, in particular the direct identification of staging of changes to the layer spacing during the process allows a better understanding of the features of the reaction and helps to explain unusual structural results (such as the structure of IPC-6 ^20^) that have been previously reported. It is clear that the interplay between the local and long-range changes needs to be understood in more detail before all the nuances of these complex reactions can be explained and the ADOR process can be used in the controlled design of novel zeolites. The *in situ* techniques described here are, of course, not limited solely to the ADOR process. Any interconversion of zeolite materials,^60^ could also be studied by these types of experiment, and the combination of local and long-range structure information could play an important part in understanding many such processes.

## Supplementary Material



## Notes

Additional information on NMR, XRD, microscopy, and the kinetic analyses. The research data supporting this publication can be accessed at 10.17630/4061c24b-82d0-48f3-9ce0-77eb3a2fbe95 ^61^ (PDF)
