# Supplementary material for: Understanding the Mechanism of Nontraditional Zeolite Synthesis Using In Situ Nuclear Magnetic Resonance Spectroscopy and X‑ray Diffraction
Source: J Am Chem Soc. 2025 Dec 22;148(1):1478–92. doi: 10.1021/jacs.5c17807 (PMC12814350; doi:10.1021/jacs.5c17807)
Supplement: Supplementary file 1 [file ja5c17807_si_001.pdf]

Understanding the Mechanism of Non-Traditional Zeolite Synthesis using *In Situ* Nuclear Magnetic Resonance Spectroscopy and X-Ray Diffraction

Nicole L. Kelly,[1] Emma A. L. Borthwick,[1] Gaynor B. Lawrence,[1] Paul S. Wheatley,[1] Arosha A. K. Karunathilake,[1] Oxana V. Magdysyuk,[1] David C. Lloyd,[1] Colan E. Hughes,[2] Kenneth D. M. Harris,[2] Russell E. Morris[1]* and Sharon E. Ashbrook[1]*

[1] *School of Chemistry, EaStCHEM and Centre of Magnetic Resonance, University of St Andrews, North Haugh, St Andrews KY16 9ST, UK*

[2] *School of Chemistry, Cardiff University, Park Place, Cardiff CF10 3AT UK*

Supporting Information

S1. Additional information for NMR experiments

S2. Additional information for PXRD and microscopy experiments

S3. Additional information on the kinetic fits

S4. References

S1. Additional information for NMR experiments

^{29}Si MAS NMR spectra of starting material and intermediates

Figure S1.1 shows ^{29}Si MAS NMR spectra of the parent zeolite Ge-UTL and two possible products of its hydrolysis, IPC-1P and IPC-2P. These latter two materials were synthesised using *ex situ* hydrolysis of Ge-UTL with 0.1 M HCl at 90 °C. The NMR spectra were acquired at 9.4 T using pulses of ~3.2 μs (ν_1 = 78 kHz) and an MAS rate of 10 kHz by averaging between 80 and 528 transients with a recycle interval of 120 s. Under the conditions described above, experimental Q^4/Q^3 ratios of ~10.6, ~2.8 and ~4.8 are seen for Ge-UTL, IPC-1P and IPC-2P, which can be compared with ideal ratios of ∞, 2.75 and 7.00, respectively. The ^{29}Si CP MAS NMR spectrum of Ge-UTL (acquired using a contact time of 25 ms by averaging 16 transients with a recycle interval of 5 s) reveals low levels of Q^3 defects present in the starting material.

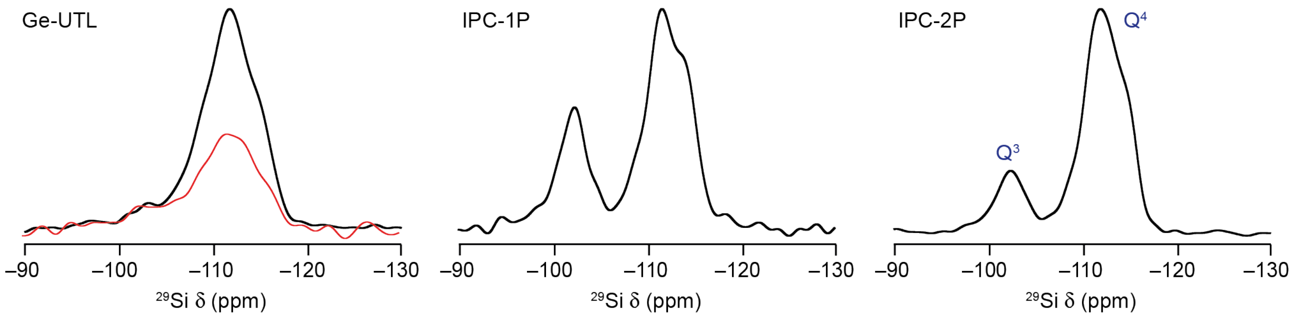

Figure S1.1. ^{29}Si (9.4 T, 10 kHz) MAS NMR spectra of Ge-UTL, IPC-1P and IPC-2P. For Ge-UTL, the corresponding CP MAS NMR spectrum is also shown in red (scaled simply to guide the eye).

^{29}Si relaxation measurements

As described in detail in Ref. S1, the ^{29}Si T_1 relaxation times for the Q^4 and Q^3 species in filtered, washed and dried samples are 12-13 s for IPC-1P and 7-9 s for IPC-2P. In the ideal case (for quantitative measurements) recycle intervals of 5 T_1 (~45-60 s) would be required; however, when studying chemical reactivity, a compromise is required between complete relaxation and the time resolution needed. The T_1 measurements quoted above were taken

at room temperature on dried samples, and relaxation is expected to be more rapid both when a hydrolysing solution is present and as the temperature increases. The relative relaxation of Q^4 and Q^3 species is very similar in each of the two materials, suggesting accurate relative ratios will be obtained even at shorter recycle intervals. It is challenging to measure T_1 accurately for the intermediate species present during the *in situ* reactions as species are evolving throughout when a solution is present. However, for the similar system studied in previous work (IPC-1P reacting with TEOS) little difference was seen in the spectral intensities for Q^4 and Q^3 Si species when recycle intervals above 15 s were used, suggesting T_1 values of < 5 s for both species. Therefore, in this work recycle intervals of 1 s and 30 s were used, with the aims of selective observation of any species in solution in the first and to extract quantitative Q^4/Q^3 ratios for the solid phase in the second.

Fitting of spectra from in situ NMR experiments

After acquisition, the interleaved ^{29}Si MAS NMR spectra were reordered to produce separate two-dimensional datasets for each of the two recycle intervals used, and each was Fourier transformed and phased. The solid-state signals cannot be fitted using single Gaussians as they result from the overlap of multiple types of distinct Si species, which restricts simple analytical fitting. Therefore, integrated intensities for the Q^n species were determined using numerical integration in MatLab 2019b using the trapz function.

To estimate the uncertainty in the measurements, integration was also performed manually in Topspin for nine spectra (three from the start, middle and later part of the reaction), each of which were processed and analysed three times and the average values determined. The difference between these average values and the MatLab 2019b values was taken as an estimate of the error. This is shown (expressed as a %) by the error bars on the plots in Figures 3 and 8 of the main text and those in the Supporting Information.

In situ NMR experiments

Figures S1.2 and S1.3 show the complete set of *in situ* ^{29}Si MAS NMR spectra acquired during the reaction of Ge-UTL with different hydrolysing solutions, using longer and shorter recycle intervals, respectively. Figure S1.4 shows the variation in the Q^4/Q^3 intensity ratio extracted from the ^{29}Si (20.0 T, 5 kHz) MAS NMR spectra shown in Figure S1.2.

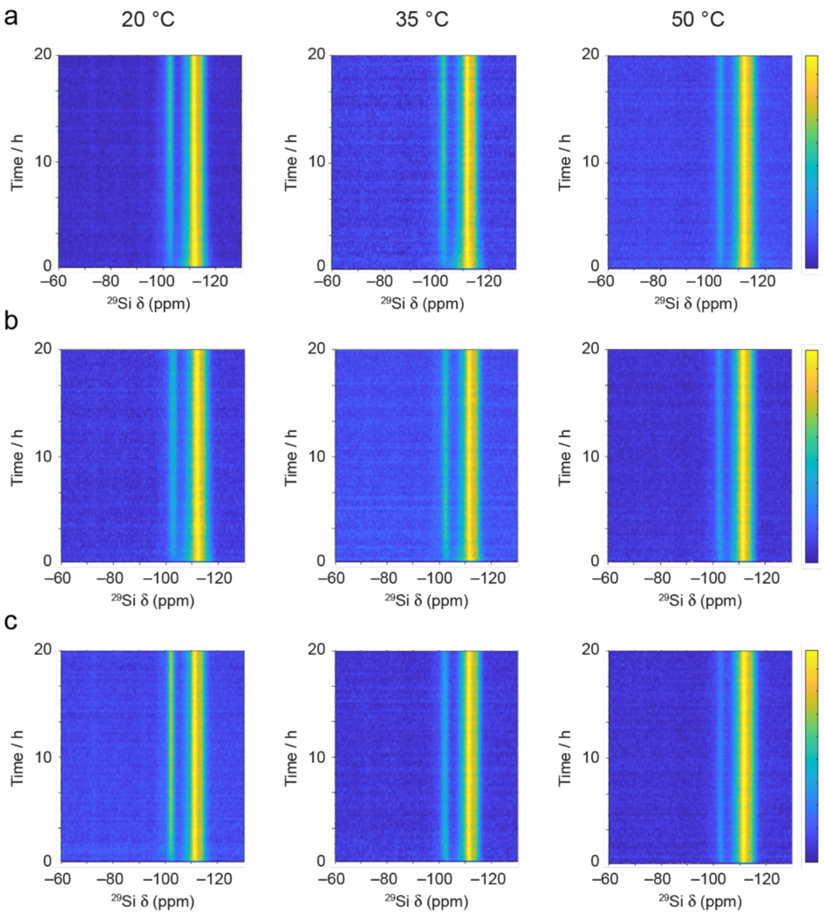

Figure S1.2. ^{29}Si (20.0 T, 5 kHz) MAS NMR spectra (shown as intensity contour plots) acquired during the *in situ* reaction of Ge-UTL with a hydrolysing solution of (a) H_2O, (b) 3 M HCl and (c) 6 M HCl, at temperatures of 20, 35 and 50 °C, with a recycle interval of 30 s.

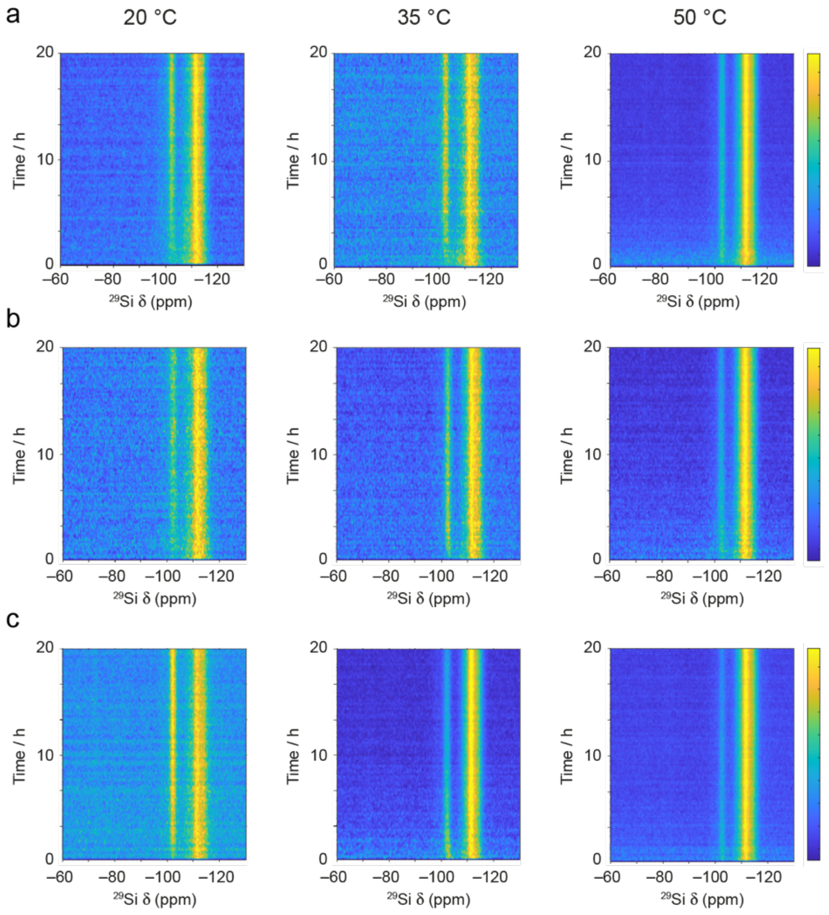

Figure S1.3. ^{29}Si (20.0 T, 5 kHz) MAS NMR spectra (shown as intensity contour plots) acquired during the *in situ* reaction of Ge-UTL with a hydrolysing solution of (a) H_2O, (b) 3 M HCl and (c) 6 M HCl, at temperatures of 20, 35 and 50 °C, with a recycle interval of 1 s.

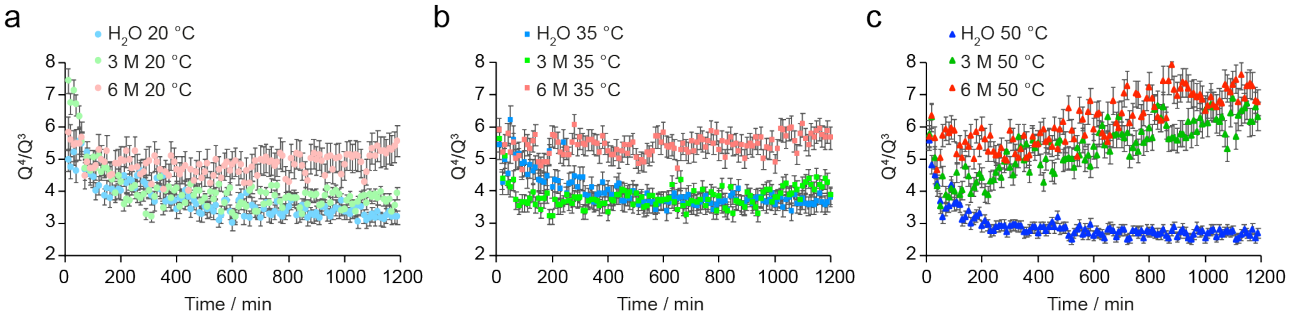

Figure S1.4. Plots showing the variation in the Q^4/Q^3 intensity ratio extracted from the ^{29}Si (20.0 T, 5 kHz) MAS NMR spectra acquired during the *in situ* reaction of Ge-UTL with (a) 20 °C, (b) 35 °C and (c) 50 °C shown in Figure S1.2. Data from reactions carried out in water, 3 M HCl and 6 M HCl are shown in blue, green and red, respectively.

The PXRD pattern was recorded for the sample recovered at the end of each *in situ* NMR experiment, as shown in Figure S1.5. Note that in most cases the PXRD pattern was acquired multiple days after the NMR experiments had been performed, leading at lower temperatures to extended reaction times. The d_{200} spacings obtained from these patterns are given in Table S1.1.

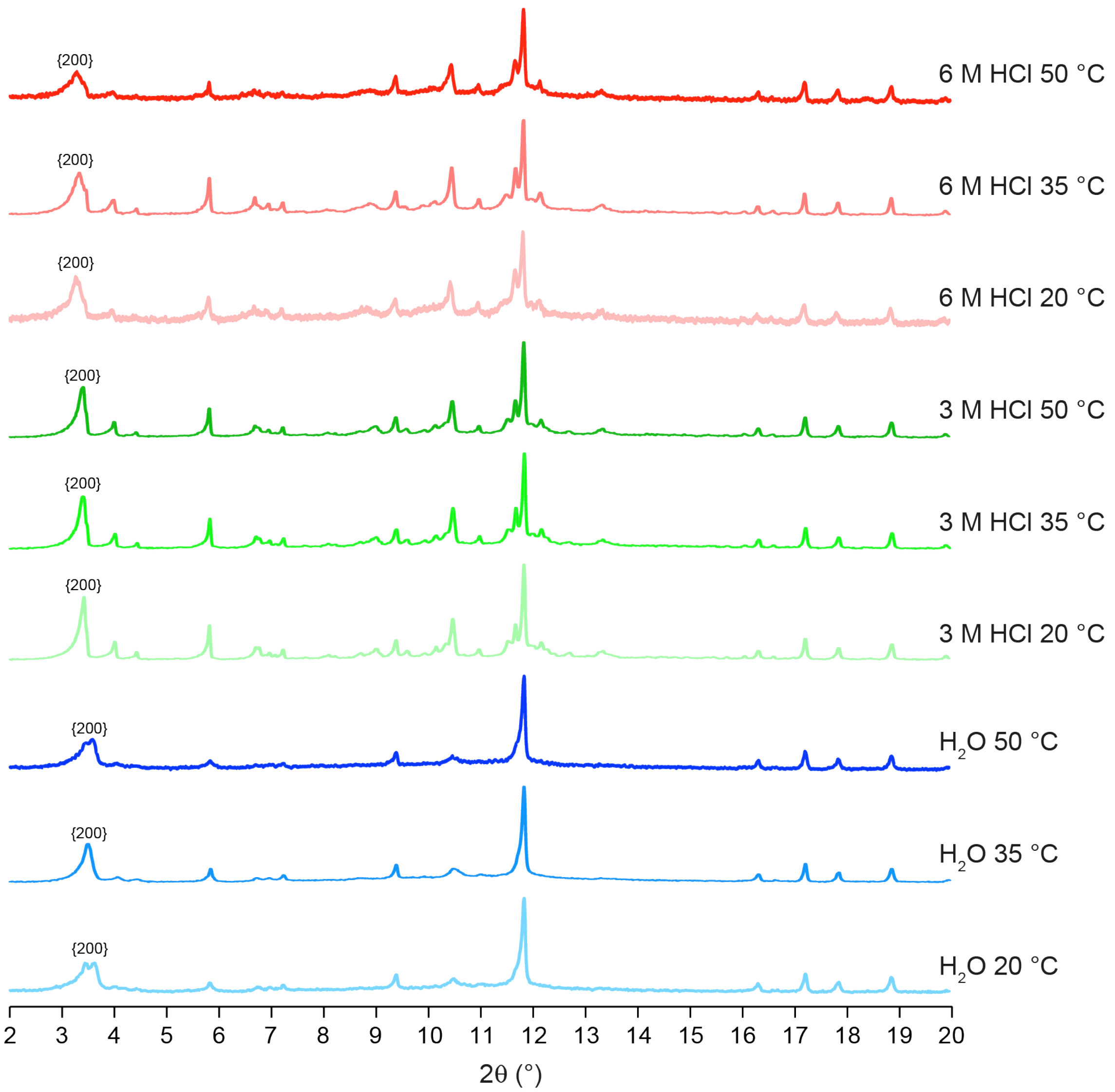

Figure S1.5. PXRD patterns (measured *ex situ*) of the final products from the *in situ* NMR experiments of the reaction of Ge-UTL under the conditions shown. The {200} peak is indicated in each case. See Section S2 for further discussion of peaks relating to GeO_2.

Table S1.1. The d_{200} spacings extracted from the PXRD patterns in Figure S1.5 for the final products of the *in situ* NMR experiments carried out under the conditions shown.

Solution	Temperature / °C	d_{200} / Å
H_2O	20	11.8
H_2O	35	11.6
H_2O	50	11.8
3 M HCl	20	11.8
3 M HCl	35	12.0
3 M HCl	50	12.0
6 M HCl	20	12.5
6 M HCl	35	12.2
6 M HCl	50	12.4

S2. Additional information for powder XRD measurements

Powder XRD of starting material and intermediates

Figure S2.1 shows powder XRD (PXRD) patterns of the parent zeolite Ge-UTL and two hydrolysis products, IPC-1P and IPC-2P, synthesised using *ex situ* hydrolysis of Ge-UTL with 0.1 M HCl at 90 °C. Patterns were acquired using a STOE STADIP instrument using a Mo X-ray tube with a primary beam monochromator ($MoK_{\alpha1}$ = 0.709 Å) at room temperature. Experimental d_{200} spacings of 14.4, 10.8 and 11.8 Å are seen for Ge-UTL, IPC-1P and IPC-2P, which can be compared with ideal values of 14.4, 10.5 and 11.7 Å, respectively.

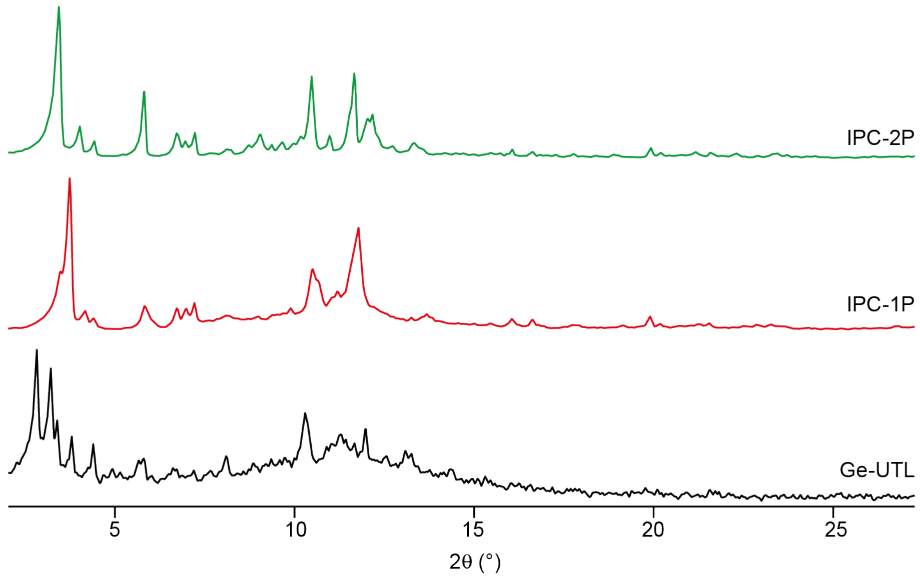

Figure S2.1. PXRD patterns of the starting Ge-UTL and typical IPC-1P and IPC-2P intermediates (synthesised by *ex situ* hydrolysis of Ge-UTL).

In situ PXRD experiments

Figure S2.2 shows the complete set of PXRD patterns acquired (shown as contour plots as a function of time) for the *in situ* reaction of Ge-UTL and a hydrolysing solution of H_2O at temperatures between 45 and 80 °C. Figure S2.3 shows contour plots of the corresponding sets of PXRD patterns for the *in situ* reactions of Ge-UTL and 3 M and 6 M HCl, at temperatures of 20, 35, 50 and 80 °C. Patterns extracted from the start, midway point and end of each reaction are shown in Figures S2.4 and S2.5, for reactions in water and in HCl,

respectively. Note that the reactions are studied for different total durations, so the times for the midway and end points will be different in each case. The PXRD pattern taken after 70 h of the *in situ* reaction of Ge-UTL with 3 M HCl at 50 °C, showing the formation of crystalline GeO_2 in the reaction, is shown in Figure S2.6.

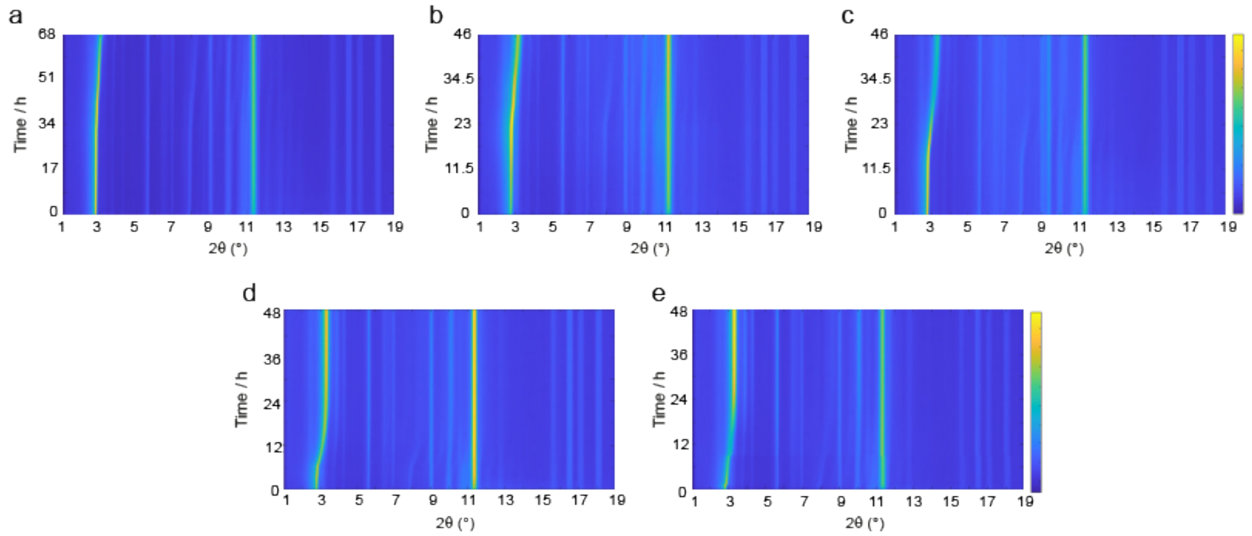

Figure S2.2. PXRD patterns (shown as intensity contour plots) acquired during the *in situ* reaction of Ge-UTL with a hydrolysing solution of H_2O at temperatures of (a) 45 °C, (b) 50 °C, (c) 55 °C, (d) 70 °C and (e) 80 °C.

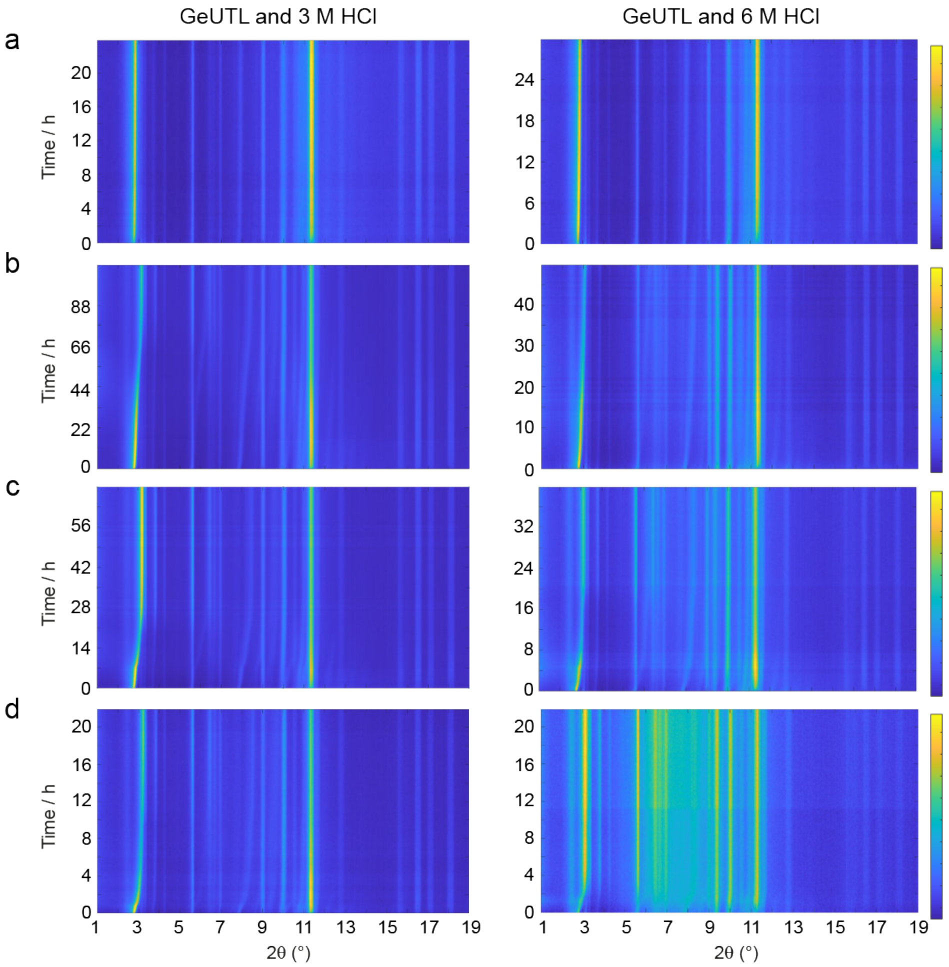

Figure S2.3. PXRD patterns (shown as intensity contour plots) acquired during the *in situ* reaction of Ge-UTL with a hydrolysing solution of (left) 3 M HCl and (right) 6 M HCl, at temperatures of (a) 20 °C, (b) 35 °C, (c) 50 °C and (d) 80 °C.

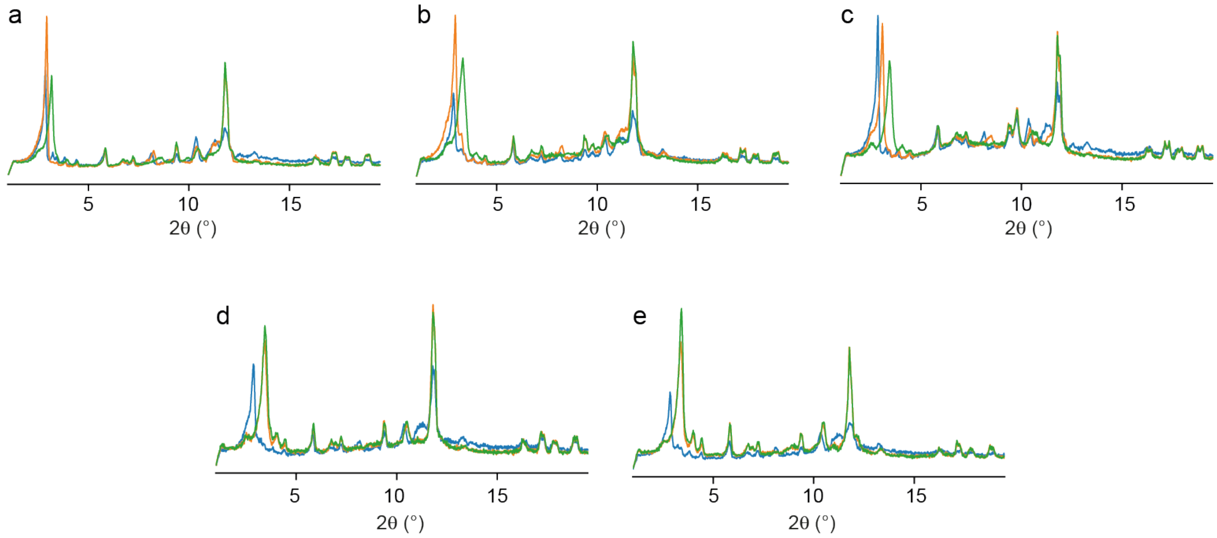

Figure S2.4. PXRD patterns recorded at different times during the *in situ* reaction of Ge-UTL with a hydrolysing solution of H_2O at temperatures of (a) 45 °C, (b) 50 °C, (c) 55 °C, (d) 70 °C and (e) 80 °C. In each case, PXRD patterns are shown at the beginning (blue), midway point (orange) and end (green) of the reaction. Note that the reactions were studied for different total durations. The full set of PXRD data recorded in each of these *in situ* experiments is shown in Figure S2.2.

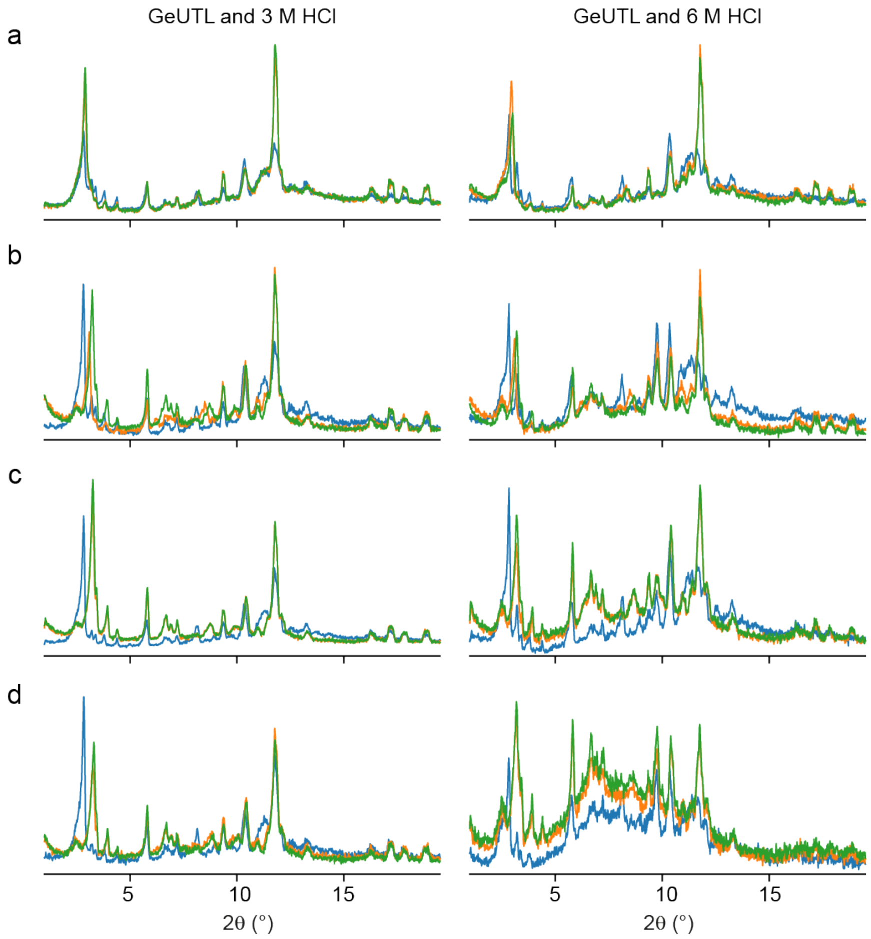

Figure S2.5. PXRD patterns recorded at different times during the *in situ* reaction of Ge-UTL with a hydrolysing solution of (left) 3 M HCl and (right) 6 M HCl, at temperatures of (a) 20 °C, (b) 35 °C, (c) 50 °C and (d) 80 °C. In each case, PXRD patterns are shown at the beginning (blue), midway point (orange) and end (green) of the reaction. Note that the reactions were studied for different total durations. The full set of PXRD data recorded in each of these *in situ* experiments is shown in Figure S2.3.

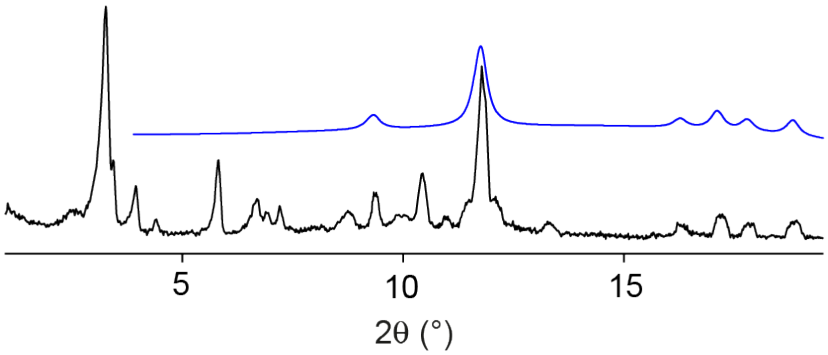

Figure S2.6. PXRD pattern (black) recorded after 70 h of the *in situ* reaction of Ge-UTL with 3 M HCl at 50 °C, shown with a simulated PXRD pattern (blue) for crystalline GeO_2.

Plots of the variation in linewidth (full width half maximum, FWHM) of the {200} reflection in PXRD patterns recorded during the *in situ* reactions of Ge-UTL with hydrolysing solutions of water and HCl for reactions carried out in water and acid are shown in Figures S2.7 and S2.8, respectively. These data are shown overlaid on plots of the corresponding change in the d_{200} spacing (shown in Figure 5 of the main text) determined from the same *in situ* PXRD data.

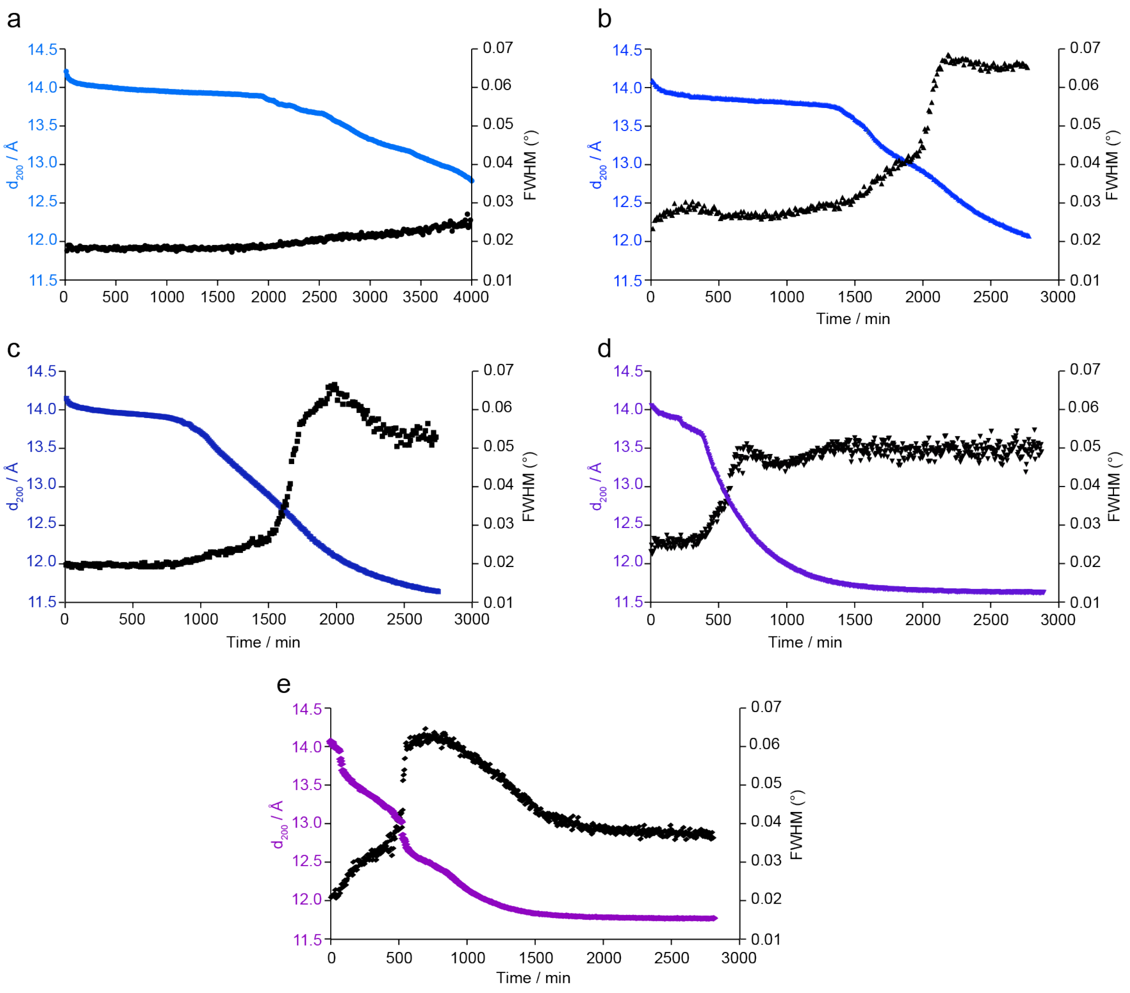

Figure S2.7. Plots of the variation in the linewidth (FWHM; shown in black) and the d_{200} spacing (shown in blue/purple) of the {200} reflection in the PXRD patterns shown in Figure S2.2, acquired during the *in situ* reaction of Ge-UTL with a hydrolysing solution of H_2O at temperatures of (a) 45 °C, (b) 50 °C, (c) 55 °C, (d) 70 °C and (e) 80 °C.

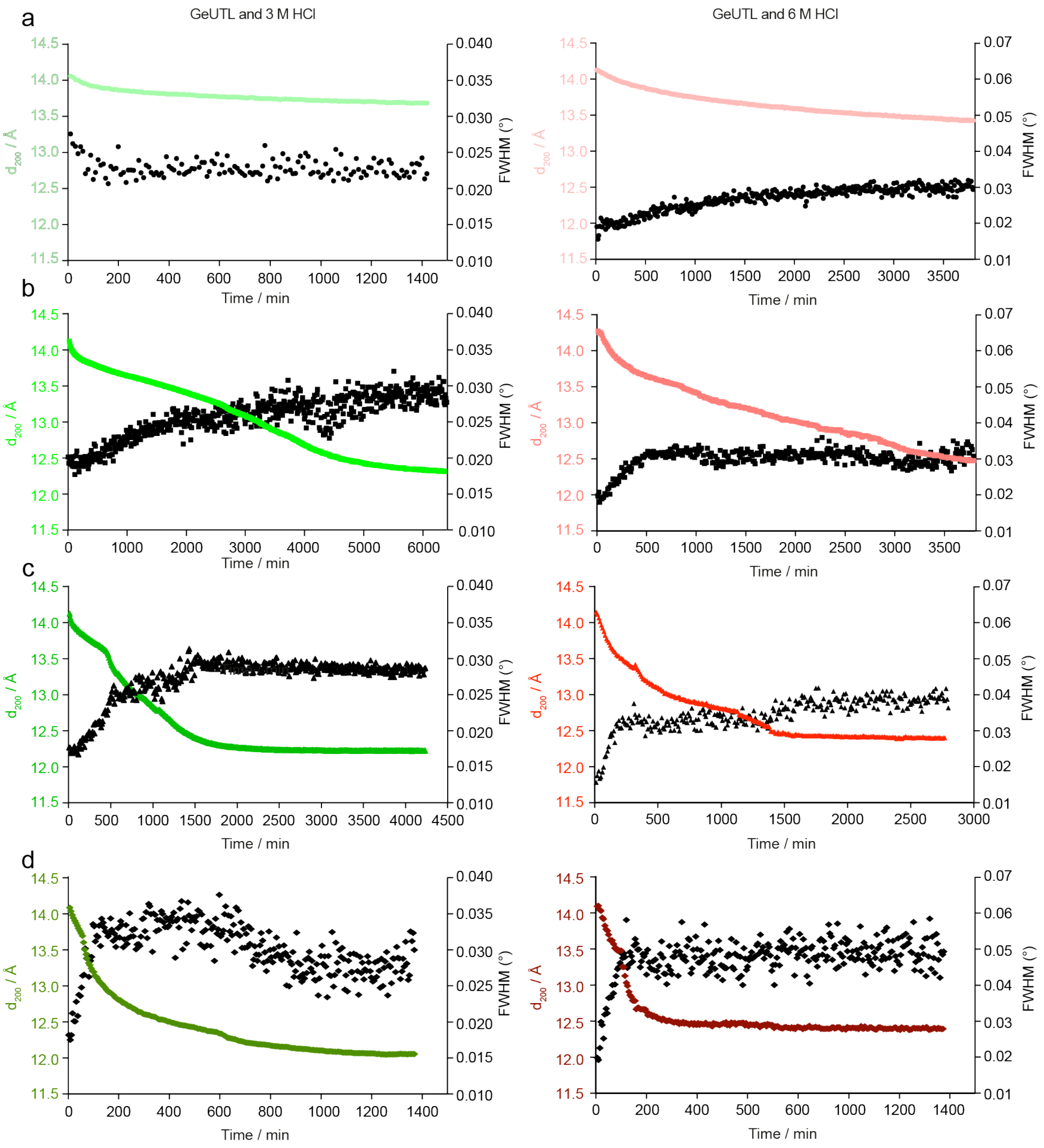

Figure S2.8. Plots of the variation in the linewidth (FWHM; shown in black) and the d_{200} spacing (shown in green/red) of the {200} reflection in the PXRD patterns shown in Figure S2.2, acquired during the *in situ* reaction of Ge-UTL with a hydrolysing solution of (left) 3 M and (right) 6 M HCl at temperatures of (a) 20 °C, (b) 35 °C, (c) 50 °C and (e) 80 °C.

Figure S2.9 shows TEM and EDS images of the ~500 nm triangular particles that are present after the *in situ* XRD reactions of Ge-UTL with 6 M HCl at 50 °C. These samples contain Si but not Ge, but unlike the IPC-2P like material they show crystallinity. This suggests they are SiO_2 resulting from the breakdown of the silicate zeolite structure itself when high reaction temperatures are used at high levels of acidity, as discussed in the main text.

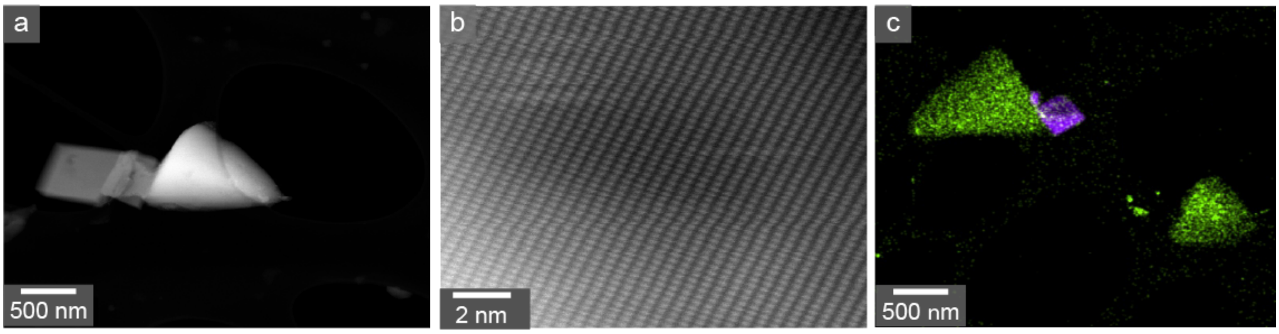

Figure S2.9. (a, b) TEM and (c) EDS images of the product of the reaction of Ge-UTL with 6 M HCl at 50 °C monitored using *in situ* XRD. The smaller cubes result from GeO_2 and the triangular particles from SiO_2 that results from breakdown of the silicate zeolite structure itself when high reaction temperatures are used at high levels of acidity.

S3. Additional information on the kinetic fits

Experimental *in situ* NMR data were analysed using an Avrami-Erofe'ev (JMAK) type kinetic approach,[S2] where the Avrami-Erofe'ev equation is

$$\mathbf{x}_t = 1 - \exp(-kt^n) \ , \qquad \text{(S3.1)}$$

where $\mathbf{x}_t$ is the relative amount of species **x** at time t, k is the rate constant and n (which can vary between 0 and 4) gives information on the dimensionality and nucleation properties of the process.

Reaction of Ge-UTL with water

Figure S3.1a shows a set of best fits from the kinetic analysis of the experimental *in situ* NMR data from the reaction of Ge-UTL with water at 50 °C, with n_{diss} fixed at the values shown and k_{diss} allowed to vary. Corresponding parameters are given in Table S3.1. In the analysis it has been assumed that only a single process takes place (disassembly), modelled by a single Avrami expression, and that this goes to completion (i.e., forming an IPC-1P like product). An initial starting Q^4/Q^3 ratio of ~10 has been assumed, as measured for the starting Ge-UTL. Better agreement between experiment and theory is obtained when $n_{diss} \approx 0.2$, and results for n_{diss} values that are lower or higher are clearly less good, giving some confidence in the robustness of the fit. Figure S3.1b shows the results from a similar kinetic analysis where both n_{diss} and k_{diss} are allowed to vary (parameters also given in Table S3.1).

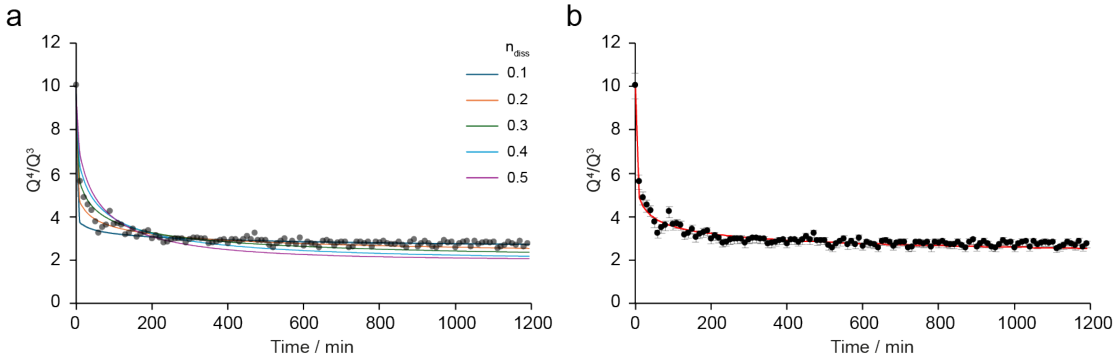

Figure S3.1. Plots showing the best fits from the kinetic analysis for the experimental ^{29}Si NMR data from the *in situ* reaction of Ge-UTL with water at 50 °C. (a) Best fits for analyses with n_{diss} fixed and k_{diss} is allowed to vary. (b) Best fits with both n_{diss} and k_{diss} allowed to vary. Corresponding kinetic parameters are given in Table S3.1.

Table S3.1. Kinetic parameters determined from analysis of data (variation in Q^4/Q^3 as a function of time) from the ^{29}Si *in situ* NMR experiments under the conditions shown. Note units of k_{diss} are $min^{-n_{diss}}$.

Solution	Temperature / °C	n_{diss}	k_{diss}	Figure
H_2O	50	0.1	1.178*	S3.1a
H_2O	50	0.2	0.657*	S3.1a
H_2O	50	0.3	0.377*	S3.1a
H_2O	50	0.4	0.224*	S3.1a
H_2O	50	0.5	0.141*	S3.1a
H_2O	50	0.204*	0.643*	S3.1b/8a
H_2O	20	0.204	0.457*	S3.2
H_2O	35	0.204	0.407*	S3.2
H_2O	20	0.197*	0.476*	8b
H_2O	35	0.159*	0.535*	8c

* Value allowed to vary in the fitting

As the disassembly process does not go to completion in the *in situ* reactions of Ge-UTL with water at 20 °C and 35 °C, analyses were initially carried out by fixing n_{diss} to the value obtained for the reaction at 50 °C (i.e., assuming the same process is taking place) and varying k_{diss}. The resulting fits are shown in Figure S3.2, with kinetic parameters given in Table S3.1. Although reasonable fits are found, slightly better fits can be obtained by letting both n_{diss} and k_{diss} vary (see Figure 8 of the main text and parameters in Table S3.1). These fits give n_{diss} close to 0.2 in each case (with the small variation likely arising from the scatter in the data), and k_{diss} values that increase from 0.476 at 20 °C to 0.643 at 50 °C.

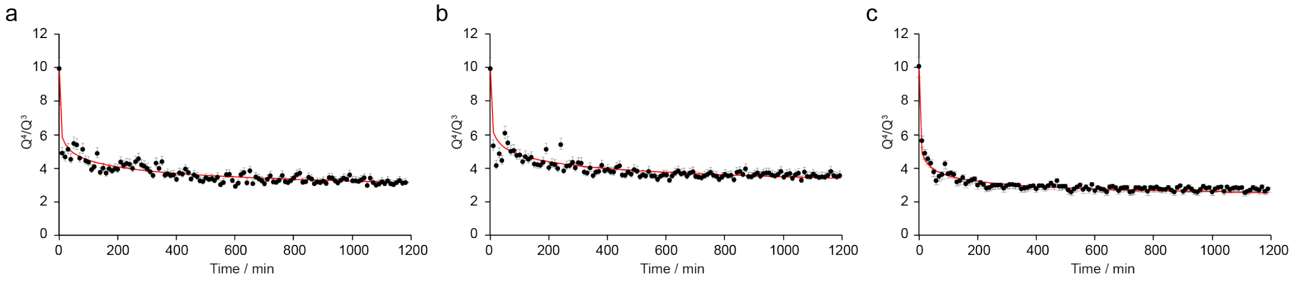

Figure S3.2. Plots showing the best fits from the kinetic analysis for the experimental NMR data from the *in situ* reaction of Ge-UTL with water at (a) 20 °C, (b) 35 °C and (c) 50 °C, with n_{diss} = 0.204 and k_{diss} allowed to vary. Corresponding kinetic parameters are given in Table S3.1.

Reaction of Ge-UTL with acid

When the hydrolysis is carried out in acidic solution both disassembly and organisation steps are expected to occur. Figure S3.3 shows the best fits from the kinetic analysis of the experimental *in situ* ^{29}Si NMR data from the reaction of Ge-UTL with 3 M and 6 M HCl at 50 °C, with the parameters for the disassembly step (n_{diss} and k_{diss}) fixed at those obtained for the corresponding reaction with water at the same temperature (Figure S3.3a), and with the parameters for the organisation step (n_{org} and k_{org}) allowed to vary. Corresponding kinetic parameters are given in Table S3.2. It is clear that the fits for the acidic hydrolyses are poor, particularly for the early stages of the reaction, suggesting that the disassembly process is distinctly different (i.e., has a different n_{diss}) as well as simply happening more rapidly. It was found not possible to vary all four kinetic parameters within the fitting as this is a under

constrained problem; when all four kinetic parameters were varied, the solutions were found to depend significantly on the initial starting conditions and to result in unphysical values of the parameters. Figure 13 in the main text shows the best fits for the reactions carried out with 3 M and 6 M HCl at all temperatures with n_{diss} and n_{org} fixed at 0.4 (as determined in previous work)[S1,S3] and with k_{diss} and k_{org} allowed to vary. Corresponding parameters are given in Table S3.2 of the Supporting Information.

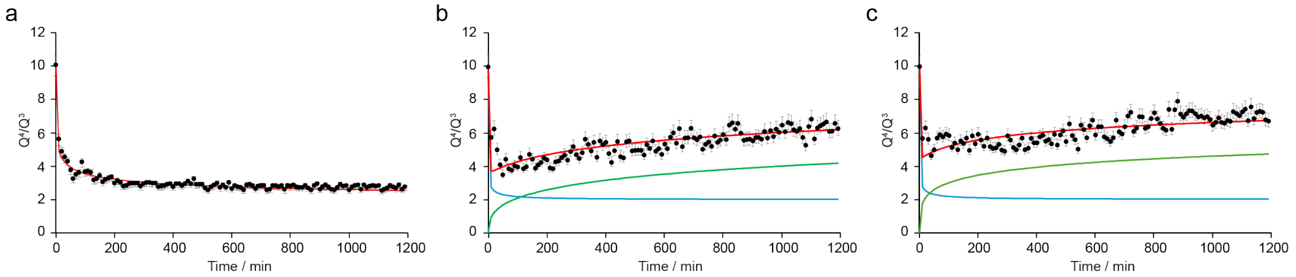

Figure S3.3. Plots showing the best fits from the kinetic analysis for the experimental ^{29}Si NMR data from the *in situ* reaction of Ge-UTL with (a) water, (b) 3 M HCl and (c) 6 M HCl at 50 °C. In (a), n_{diss} and k_{diss} were both allowed to vary, and in (b, c) n_{diss} and k_{diss} were both fixed at the values determined in (a) and n_{org} and k_{org} were allowed to vary. Corresponding kinetic parameters are given in Table S3.2.

Table S3.2. Kinetic parameters determined from analysis of data (variation in Q^4/Q^3 as a function of time) from the *in situ* NMR experiments under the conditions shown. Note units of k_i are min^{-n_i}.

Solution	Temperature / °C	n_{diss}	k_{diss}	n_{org}	k_{org}	Figure
H_2O	50	0.204*	0.643*			S3.1b/8a
3 M HCl	50	0.204	0.643	0.381*	0.013*	S3.3a
6 M HCl	50	0.204	0.643	0.283*	0.049*	S3.3a
3 M HCl	20	0.4	0.038*	0.4	0.0025*	13a
3 M HCl	35	0.4	0.067*	0.4	0.004*	13b
3 M HCl	50	0.4	0.094*	0.4	0.010*	13c
6 M HCl	20	0.4	0.051*	0.4	0.006*	13d
6 M HCl	35	0.4	0.052*	0.4	0.009*	13e
6 M HCl	50	0.4	0.076*	0.4	0.014*	13f

* Value allowed to vary in the fitting

For each of the reactions carried out under acidic conditions, Figure S3.4a shows the point at which the organisation process becomes the more dominant contributor to the changes in Q^4/Q^3 (corresponding to the point at which the green line crosses the blue line in Figure 13). Figure S3.4b shows the minimum and final Q^4/Q^3 values for each of the acidic hydrolyses, reflecting the occurrence of both a rapid disassembly step and a slower organisation step, and with the rate of the latter process increasing with both acidity and temperature.

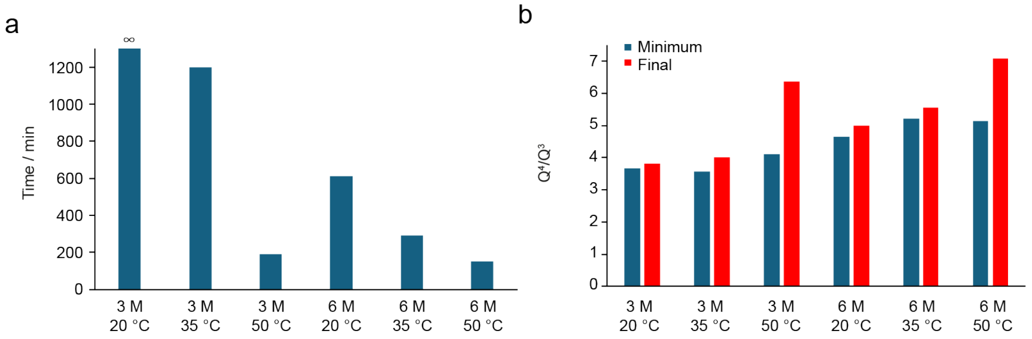

Figure S3.4. Plots showing (a) the time at which the organisation process becomes the more dominant contributor to the changes in Q^4/Q^3 and (b) the minimum and final values of Q^4/Q^3 for the ^{29}Si *in situ* NMR reactions carried out in acidic conditions. Experimental data and corresponding fits are shown in Figure 13 of the main text.

S4. References

S1. Kelly, N. L.; Borthwick, E. A. L.; Lawrence, G. B.; Wheatley, P. S.; Hughes, C. E.; Harris, K. D. M.; Morris, R. E.; Ashbrook, S. E. Exploiting *in situ* NMR spectroscopy to understand non-traditional methods for zeolite synthesis. *Chem. Sci.* **2025**, *16*, 4245-4255. DOI: 10.1039/D4SC07931K.

S2. Shirzad, J.; Viney, C. A critical review on applications of the Avrami equation beyond materials science. *J. Roy. Soc. Interface* **2023**, *20*, 20230242. DOI: 10.1098/rsif.2023.0242.

S3. Henkelis, S. E.; Mazur, M.; Rice, C. M.; Wheatley, P. S.; Ashbrook, S. E.; Morris, R. E. Kinetics and Mechanism of the Hydrolysis and Rearrangement Processes within the Assembly–Disassembly–Organization–Reassembly Synthesis of Zeolites. *J. Am. Chem. Soc.* **2019**, *141*, 4453-4459. DOI: 10.1021/jacs.9b00643.
